# Weekends affect mortality risk and chance of discharge in critically ill patients: a retrospective study in the Austrian registry for intensive care

**DOI:** 10.1186/s13054-017-1812-0

**Published:** 2017-09-07

**Authors:** Paul Zajic, Peter Bauer, Andrew Rhodes, Rui Moreno, Tobias Fellinger, Barbara Metnitz, Faidra Stavropoulou, Martin Posch, Philipp G. H. Metnitz

**Affiliations:** 1 Division of General Anesthesiology, Emergency and Intensive Care Medicine, Department of Anesthesiology and Intensive Care Medicine, Medical University of Graz, Auenbruggerplatz 29, A-8036 Graz, Austria; 2 Centre for Medical Statistics, Informatics, and Intelligent Systems, Medical University of Vienna, Vienna, Austria; 3 St George’s University Hospitals NHS Foundation Trust, St George’s University of London, London, UK; 4 Unidade de Cuidados Intensivos Neurocríticos, Hospital de São José, Centro Hospitalar de Lisboa Central, Lisbon, Portugal; 5 Austrian Centre for Documentation and Quality Assurance in Intensive Care, Vienna, Austria

**Keywords:** Critical care, Mortality, Patient discharge, Quality of health care

## Abstract

**Background:**

In this study, we primarily investigated whether ICU admission or ICU stay at weekends (Saturday and Sunday) is associated with a different risk of ICU mortality or chance of ICU discharge than ICU admission or ICU stay on weekdays (Monday to Friday). Secondarily, we analysed whether weekend ICU admission or ICU stay influences risk of hospital mortality or chance of hospital discharge.

**Methods:**

A retrospective study was performed for all adult patients admitted to 119 ICUs participating in the benchmarking project of the Austrian Centre for Documentation and Quality Assurance in Intensive Care (ASDI) between 2012 and 2015. Readmissions to the ICU during the same hospital stay were excluded.

**Results:**

In a multivariable competing risk analysis, a strong weekend effect was observed. Patients admitted to ICUs on Saturday or Sunday had a higher mortality risk after adjustment for severity of illness by Simplified Acute Physiology Score (SAPS) 3, year, month of the year, type of admission, ICU, and weekday of death or discharge. Hazard ratios (95% confidence interval) for death in the ICU following admission on a Saturday or Sunday compared with Wednesday were 1.15 (1.08–1.23) and 1.11 (1.03–1.18), respectively. Lower hazard ratios were observed for dying on a Saturday (0.93 (0.87–1.00)) or Sunday (0.85 (0.80–0.91)) compared with Wednesday. This is probably related to the reduced chance of being discharged from the ICU at the weekend (0.63 (0.62–064) for Saturday and 0.56 (0.55–0.57) for Sunday). Similar results were found for hospital mortality and hospital discharge following ICU admission.

**Conclusions:**

Patients admitted to ICUs at weekends are at increased risk of death in both the ICU and the hospital even after rigorous adjustment for severity of illness. Conversely, death in the ICU and discharge from the ICU are significantly less likely at weekends.

**Electronic supplementary material:**

The online version of this article (doi:10.1186/s13054-017-1812-0) contains supplementary material, which is available to authorized users.

## Background

Results from recent studies suggesting that increased mortality is associated with weekend admission to National Health Service (NHS) hospitals in the United Kingdom—the so-called “weekend effect”—have prompted intensive discussions in both the scientific community and the public [1–3]. In studies like these, adjustment for severity of illness is paramount, since the case mix may differ substantially between weekends and weekdays [4]. This adjustment is of utmost importance in critically ill patients, which may explain why a recent study, focused on patients in intensive care units (ICUs) in the NHS, found no discernible weekend effects following emergency admission to the ICU [5].

Because there is no generally agreed-on methodological approach, the existing body of evidence is inconsistent. While increased risk of death following weekend ICU admission has been found in some studies [6–8], other studies have failed to demonstrate any weekend effects [9–13] following adjustment for severity of illness. Yet a meta-analysis based on data available in 2010 has concluded that weekend ICU admissions are associated with increased risk of death [14]. As the available evidence is conflicting and confounded by several factors, additional high-quality data are required to address the question of whether there are “weekend effects” in ICU patients. Furthermore, it needs to be clarified whether these supposed effects affect mortality risk only, and whether the admission day represents the only influencing factor.

To specifically assess the impact of intensive care upon patient outcomes, it is prudent to focus on ICU mortality following adjustment for baseline risk of death and type of admission as the primary variable of interest. Death in the ICU may obviously be preceded by discharge or transfer from the ICU at any given time point. In this setting, competing risk analysis may be the methodological approach of choice [15]. In this study, we therefore primarily investigate whether ICU admission or ICU stay at weekends (Saturday and Sunday) is associated with a different risk of ICU mortality or chance of ICU discharge than ICU admission or ICU stay on weekdays (Monday to Friday). Secondarily, we analyse whether weekend ICU admission or ICU stay influences the risk of hospital mortality or chance of hospital discharge.

## Methods

The Austrian Centre for Documentation and Quality Assurance in Intensive Care (ASDI) is a non-profit organisation which has established a multicentre database containing anonymised data on patients admitted to ICUs in Austria (Additional file 1: Table S1). The data set is described in detail elsewhere [16]. The prospectively collected data include: sociodemographic data, such as age, sex, and chronic conditions; reason for admission, recorded according to a predefined list of medical and surgical diagnoses [17]; severity of illness, as measured by either the Simplified Acute Physiology Score (SAPS) II (used until 2011) [18] or the SAPS 3 (used since 2012) [19, 20]; level of provided care, as measured by the Simplified Therapeutic Intervention Scoring System (TISS-28) [21]; length of ICU and hospital stay; and outcome data, including survival status at ICU and hospital discharge. Since no additional interventions were performed, the need for informed consent was waived by the institutional review board.

### Statistical analysis

The main analysis was conducted using the Fine and Gray proportional subdistribution hazards model [22]. Competing events of interest were ICU mortality and ICU discharge within 30 days. Patients staying in the ICU for more than 30 days were censored, because 30-day mortality is a widely accepted outcome measure, the model is likely to fit better when applied over a limited time interval only, and previous studies on the same topic did so [1]. Sensitivity analyses without censoring after 30 days were performed. All analyses were conducted according to Beyersmann et al. [23] with R version 3.3.1 and the package survival version 2.39-4.

The following variables were used as risk factors for modelling death in the ICU or ICU discharge: weekday of admission, weekday of event (death or discharge), SAPS 3, year of admission, month of admission, type of admission as outlined in the SAPS 3 [19], and centre (ICUs as fixed effects). The weekday of death or discharge was modelled by a time-dependent covariate. Wednesday was chosen as the reference day. The survConcordance function [24] was used to evaluate the calibration of the model. Additionally, we assessed the impact of the inclusion of twofold interactions between the variables admission day, type of admission, and SAPS 3 as well as the reasons for admission. Proportionality of hazards was investigated using an interaction term between weekday of admission and time to assess a possible influence of weekday of admission on early or late mortality.

Secondary analysis consisted of investigating possible weekend effects on hospital mortality and hospital discharge following ICU admission. The model was built upon the variables already described.

Sensitivity analyses were performed to assess the magnitude of possible weekend effects in the following subgroups: patients admitted to ICUs that reported more than 99% of hospital outcomes; only readmissions during the same hospital stay; and patients in the first, second, or third tertile of the SAPS 3. The main analysis was repeated by fitting Cox proportional hazards models for the cause-specific hazards in the competing risk setting on the same sets of data, based on different risk sets of patients.

Data are generally presented as median and interquartile ranges (IQR) or absolute number (*n*) and percentage (%) unless specified otherwise.

## Results

All patients admitted to 119 participating ICUs between 1 January 2012 and 31 December 2015 (*n* = 167,425) were included in the study. This time period was chosen because the risk adjustment system was changed from SAPS II to SAPS 3 at the beginning of 2012. We considered the use of multiple risk adjustment systems to be inadequate for this study.

Patients < 18 years of age (*n* = 1762) or with missing age data (*n* = 31) were excluded from the analysis. For patients who were admitted more than once (*n* = 14,297), only the first admission was included. Patients without documented ICU admission or discharge dates or outcome (*n* = 67) were excluded. A total of 151,268 patients were available for the main analysis. For the secondary analysis, patients with missing hospital outcome (*n* = 1584) or missing discharge dates from the hospital (*n* = 2287) were also excluded, leaving 147,397 patients.

A total of 25,838 (17.1%) patients were referred to an ICU on Saturday or Sunday. In total, 86,564 (57.2%) patients were male and the median (IQR) age was 68 (54–77) years. Neither age nor the male/female distribution varied noticeably between days of the week (Table 1).

**Table 1:** Overall and weekday-specific patient characteristics

	Weekday of admission							
Variable	Total	Monday	Tuesday	Wednesday	Thursday	Friday	Saturday	Sunday
Patient admissions, *n*	151,268	24,367	25,866	25,810	25,312	24,075	13,279	12,559
Age (years), median (IQR)	68 (54–77)	68 (55–77)	67 (55–77)	67 (55–76)	67 (55–76)	68 (55–77)	67 (52–78)	68 (52–78)
Male sex, *n* (%)	86,564 (57.2%)	13,920 (57.1%)	14,778 (57.1%)	14,648 (56.8%)	14,456 (57.1%)	13,807 (57.4%)	7745 (58.3%)	7210 (57.4%)
SAPS 3, median (IQR)	44 (34–56)	43 (34–55)	42 (33–54)	42 (33–54)	42 (33–54)	43 (34–55)	48 (37–61)	48 (37–61)
TISS 28 score, median (IQR)								
Total, per patient	73 (46–165)	70 (46–147)	69 (46–145)	68 (45–155)	69 (46–168)	84 (48–169)	89 (44–206)	81 (42–192)
On admission day	29 (23–35)	29 (23–35)	29 (23–35)	29 (23–35)	29 (23–35)	29 (23–35)	29 (21–36)	28 (21–36)
ICU LOS (days), median (IQR)	3 (2–6)	3 (2–5)	3 (2–5)	3 (2–6)	3 (2–6)	3 (2–6)	3 (2–7)	3 (2–6)
LOS before ICU admission > 2 days, *n* (%)	51451 (34.5%)	9491 (39.5%)	8322 (32.7%)	9308 (36.7%)	9144 (36.7%)	9081 (38.4%)	3028 (23.0%)	3077 (24.7%)
Type of admission, *n* (%)								
Medical	55,356 (36.6%)	8484 (34.8%)	8505 (32.9%)	8713 (33.8%)	8401 (33.2%)	8082 (33.6%)	6682 (50.3%)	6489 (51.7%)
Unscheduled surgery	23,180 (15.3%)	3239 (13.3%)	3452 (13.4%)	3480 (13.5%)	3609 (14.3%)	3802 (15.8%)	2968 (22.4%)	2630 (20.9%)
Scheduled surgery	49,237 (32.6%)	8990 (36.9%)	10,063 (38.9%)	9750 (37.8%)	9469 (37.4%)	8399 (34.9%)	1329 (10.0%)	1237 (9.9%)
Unspecified surgery	16,287 (10.8%)	2541 (10.4%)	2707 (10.5%)	2696 (10.5%)	2652 (10.5%)	2618 (10.9%)	1561 (11.8%)	1512 (12.0%)
Not available	7208 (4.8%)	1113 (4.6%)	1139 (4.4%)	1171 (4.5%)	1181 (4.7%)	1174 (4.9%)	739 (5.6%)	691 (5.5%)
ICU mortality, *n* (%)	13,887 (9.2%)	2143 (8.8%)	2118 (8.2%)	2073 (8.0%)	1991 (7.9%)	2134 (8.9%)	1740 (13.1%)	1688 (13.4%)
Hospital mortality, *n* (%)	20,537 (13.7%)	3173 (13.2%)	3137 (12.3%)	3159 (12.4%)	3037 (12.1%)	3209 (13.5%)	2423 (18.5%)	2399 (19.4%)

*SAPS* Simplified Acute Physiology Score, *TISS* Therapeutic Intervention Scoring System, *LOS* length of stay

Severity of illness and reasons for admission varied noticeably between weekends and weekdays. Median (IQR) SAPS 3 was 44 (34–56); the lowest values were found from Tuesday to Thursday (42 (33–54)), and the highest on Saturday and Sunday (48 (37–61)). Overall, the documented type of admission to the ICU was “medical” in 36.6% of patients and “scheduled surgery” in 32.6%. On Saturdays and Sundays, however, 50.3% and 51.7% of patients were documented as a “medical” type of admission, respectively. Conversely, “scheduled surgery” was recorded as the type of admission in only 10.0% and 9.9% of patients on Saturdays and Sundays, respectively.

A total of 13,887 (9.2%) patients died in the ICU, and 137,381 (90.8%) were discharged. Overall, 20,537 (13.7%) hospital deaths and 129,147 hospital discharges were observed. Both unadjusted hospital and ICU mortality differed between weekdays and weekends. Eight per cent of patients admitted on a Wednesday died in the ICU, while 13.1% of patients admitted on Saturdays and 13.4% of patients admitted on Sundays died in the ICU. A total 12.4% of patients admitted to the ICU on Wednesday died in hospital; 18.5% of patients admitted to the ICU on Saturdays and 19.4% of patients admitted on Sundays died during their hospital stay. The observed-to-expected (O/E, (95% CI)) ratio for hospital mortality was 0.71 (0.69–0.73) on Wednesdays compared with 0.78 (0.75–0.80) on Saturdays and 0.79 (0.77–0.82) on Sundays. Table 1 presents detailed patient demographics and outcome data. Additional file 1: Table S2 describes reasons for admission in depth.

Patients admitted to the ICU at weekends had higher summative TISS-28 scores than patients admitted during the week, yet there was no discernible difference in these scores between individual days of admission (Table 1). There was significant variation in the frequencies of several key procedures, however, such as placement of peripheral arterial lines and central venous catheters (Additional file 1: Table S3).

### Main analysis

**Fig. 1:**
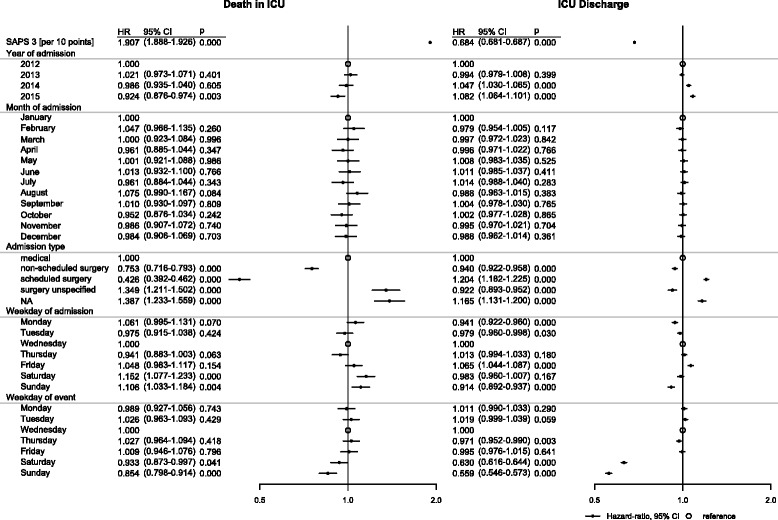
Adjusted subdistribution HR, 95% CI and *p* values for ICU mortality and ICU discharge within 30 days (*n* = 151,268). CI confidence interval, HR hazard ratio, ICU intensive care unit, SAPS Simplified Acute Physiology Score

Findings from the descriptive analysis regarding mortality were confirmed in multivariable competing risk analysis concerning the outcomes “death in the ICU” and “discharge from the ICU” within 30 days (Fig. 1).

The weekday of admission exerted a significant influence on the risk of death in the ICU. Adjusted subdistribution HRs (95% CI) for ICU mortality were 1.15 (1.08–1.23) and 1.11 (1.03–1.18), respectively, for patients admitted to an ICU on Saturday or Sunday as compared with Wednesday (Fig. 1). Chance (hazard) of ICU discharge also varied with the day of ICU admission: patients admitted on Fridays had the highest chance of discharge (HR 1.07 (95% CI 1.04–1.09)), whereas adjusted HRs for ICU discharge were significantly lower when admission was on Sunday, Monday, or Tuesday as compared with Wednesday (Fig. 1).

Conversely, the risk of dying in the ICU during weekends was significantly reduced; HRs (95% CI) for death in the ICU on Saturdays and Sundays were 0.93 (0.87–1.00) and 0.85 (0.80–0.91), respectively, as compared with Wednesday (Fig. 1). Chances of ICU discharge during weekends were also significantly lower compared with weekdays; HRs (95% CI) for discharge from the ICU were 0.63 (0.62–0.64) on Saturdays and 0.56 (0.55–0.57) on Sundays.

Risk of death in the ICU varied greatly between types of admission. Patients admitted to the ICU in the group “scheduled surgery” were at the lowest risk of ICU death (HR 0.43 (95% CI 0.39–0.46)) compared with the reference group of “medical” admission. Admission for “unscheduled surgery” was also associated with lower risk (HR 0.75 (95% CI 0.72–0.79)). A highly significant association between the SAPS 3 and mortality risk was observed (HR 1.91 (95% CI 1.89–1.93) per 10 SAPS 3 points).

The chosen explanatory variables allowed for good prediction (*C*-index = 0.863). Neither the inclusion of a quadratic term for SAPS 3 into the model nor the omission of the ICU as a fixed effect changed the results in a noticeable way. We added the time-dependent covariable “weekday of admission × time” to the model to check the proportional hazards assumption. This influence variable did not contribute significantly.

After the inclusion of interaction terms into the main model, HRs (95% CI) for death in the ICU were elevated: 1.21 (1.07–1.36) for admission on Saturday and 1.18 (1.05–1.34) for admission on Sunday (Additional file 1: Table S4). In this extended model, a noticeable interaction was identified. Weekend admissions of patients with the type of admission “scheduled surgery” were associated with increased hazards for death in the ICU compared with admissions on Wednesdays. HRs (95% CI) for the interaction between the type of admission “scheduled surgery” and the weekday of admission were 1.56 (1.14–2.14) for admission on Saturday and 1.45 (1.03–2.04) for admission on Sunday (Additional file 1: Table S2).

### Secondary analysis

Results for hospital mortality and hospital discharge (Fine and Gray model) were practically identical to the aforementioned findings. Adjusted HRs (95% CI) for hospital mortality were 1.15 (1.08–1.23) for ICU admission on Saturday and 1.11 (1.03–1.18) for ICU admission on Sunday. ICU admission on Sunday or Monday was associated with the lowest chances of hospital discharge. Risk of in-hospital death and chance of hospital discharge at weekends were also significantly lower than during the week (Additional file 1: Table S5).

### Sensitivity analyses

Findings in subgroup analyses confirmed our results’ stability. Weekend effects were almost identical in patients admitted to ICUs that reported more than 99% of hospital outcomes (*n* = 113,161) (Additional file 1: Table S6). Weekend effects were reproduced in all three tertiles of the SAPS 3 (Additional file 1: Table S7). The effects were present in all admission-type subgroups except for unscheduled surgery (Additional file 1: Table S9). When the Cox proportional hazards model was applied to the main analysis, significant weekend effects were found, although they were slightly less pronounced; concordance was 0.846 (Additional file 1: Table S10). ﻿Results from the model without censoring at 30 days did not differ significantly from the main model's results. (﻿Additional file Additional file 1: Table S11)

The inclusion of interaction terms of weekday of admission and time did not indicate any noticeable time dependency of the effect on mortality apart from a slight tendency for higher short-term mortality when admitted on Saturday (*p* = 0.03). An accentuated tendency towards earlier discharge when admitted on Friday or Saturday was observed (data not shown).

## Discussion

Our findings demonstrate that there are weekend effects in Austrian ICUs. Admission to an ICU on Saturday or Sunday was associated with both increased risk of ICU and hospital mortality and a reduced chance of ICU and hospital discharge. These findings were stable in all models, although we applied rigorous risk adjustment using the well-established SAPS 3 risk adjustment model [19, 20, 25]. Adjustment for baseline risk of death was imperative [26], as the case mix admitted to ICUs on weekends differs noticeably from admissions during the week.

These weekend effects, however, did not result in an increased risk of dying in the ICU during weekends. Mortality rates in ICUs and hospitals were actually lower on Saturdays and Sundays. Moreover, discharge from the ICU and the hospital was substantially less likely at weekends than during the week. The competing risk analysis itself offers a possible explanation for these findings. Because of the low chance of discharge at weekends, patients tended to remain in the ICU over the weekend regardless of their physiologic status. This would result in a lower risk of death at weekends.

The statistical validity of the models used can be inferred from the good results in prediction and the fairly identical results in the Fine and Gray model for subdistribution hazards and the well-established Cox proportional hazards model fitted for competing risks. A major strength of this study lies in the statistical analysis based on the competing risks concept, which models transitions in patient status (in ICU, discharged, dead). The observed concordance coefficient for the main analysis is satisfactory in heterogeneous populations such as the critically ill [27, 28].

The clinical validity of our findings relies on the SAPS 3 risk adjustment tool’s ability to adequately correct for patients’ individual risk of death. If characteristics negatively influencing outcomes were more prevalent in patients admitted at weekends and remained unadjusted for, an increased risk of death could falsely be associated with weekend admission instead of the patients’ characteristics. For this reason, we conducted detailed sensitivity analyses to assess both statistical and clinical validity.

We stratified the study cohort by tertiles of the SAPS 3 to evaluate whether the effects observed targeted specific subpopulations only. Similar effects were found, however, in all three tertiles (Additional file 1: Table S5). We can thus rule out the possibility that our results are attributable to high or low acuity bias. Additional sensitivity analyses included only patients admitted to ICUs with excellent reporting characteristics (more than 99% of all patients included in the study cohort) to minimise the risk of reporting bias (see Limitations). Weekend effects were the same in this subgroup (Additional file 1: Table S4). Weekend effects were observed when we analysed readmissions to the ICU during the same hospital stay (Additional file 1: Table S8). The results’ reliability is backed by their consistency over various subgroups of patients. The completeness of reporting in the participating ICUs is another strength of our study and is due to Austrian healthcare legislation that requires reporting of key items for all admitted patients before ICU costs are reimbursed.

Patients referred to ICUs at weekends were more likely to be admitted after emergency surgery and exhibited a higher severity of illness as measured by the SAPS 3. Increased risk of death after weekend ICU admission was not observed in patients admitted after unscheduled surgical procedures, whereas weekend effects were clearly identifiable in both subgroups of patients admitted to ICU after scheduled surgery or due to medical conditions. These findings are in concordance with previous findings from other studies that unscheduled surgery outside regular working hours is not associated with increased mortality [29–31].

In fact, scheduled surgeries are rare on weekends compared with weekdays. A higher risk of death following weekend procedures could therefore be due to a lack of experienced staff [32, 33] or insufficient resources. It could be speculated that the quality of necessary interventions provided for critically ill patients outside the ICU might influence overall outcome, affecting some patients more than others. Previous studies have demonstrated differences in outcomes following critical procedures; for example, in patients with acute myocardial infarction and stroke [34, 35].

If the higher risk of death following weekend ICU admission cannot be explained by case mix alone, the reasons for this effect need to be identified. Because of the complexity of providing critical care and the retrospective nature of this study, we are unable to give detailed answers. Possible explanations for worse outcomes in patients admitted to ICUs at weekends involve both structures and processes, such as inadequate staffing or increased workload. While high workloads on weekends were demonstrated in other studies [36], we have no direct data about physicians’ workload or ICU staffing during the observation period. The TISS-28 allows us, however, to evaluate nursing workload and the use of different sets of interventions and other measures.

Analysing these data, we found that, for all patients in the ICU, fewer “specific interventions” (according to the TISS-28) were performed on Saturdays and Sundays compared with the rest of the week (Additional file 1: Table S3). For patients admitted at weekends, however, “specific interventions” were performed at a higher rate both in and outside the ICU. These findings seem plausible, taking into account the higher severity of illness exhibited by patients admitted to ICUs on weekends. However, patients admitted to the ICU at weekends were surprisingly less likely to receive several key treatments on the day of admission, such as “central venous catheters”, “peripheral arterial lines”, and “lung function-improving treatments”.

### Limitations

This study’s findings are based on a retrospective analysis of data queried from a prospectively gathered database using a multivariable competing risk model for time-until-event data. The study is therefore subject to all limitations that apply to this study type. Documentation and coding are the responsibility of individual health care providers and may be incomplete, especially if data input is not required by law or local policy. Non-ICU data and information on decision-making (e.g. termination of care) may be fragmentary, since they are not in the focus of the ASDI database. Quality of documentation may vary between days of the week due to differences in workload [37]. Patient heterogeneity and variations in case mix between weekdays may contribute to bias affecting the analysis. We sought to adjust for these limitations as described, yet our adjustments rely upon the validity of the SAPS 3 risk stratification tool. Any limitations of this scoring system may also apply to this study. Findings from this study are not necessarily generalisable to other countries’ health systems.

## Conclusion

In summary, our study yielded several key findings. First, the case mix that healthcare professionals were confronted with at weekends was distinctly different from that seen during the week. Patients referred to the ICU at weekends were sicker than those admitted during the week, whilst those already present in the ICU would otherwise be discharged earlier. Second, ICU admission at weekends is associated with a higher risk of death in the ICU and the hospital in the subgroups of scheduled surgery and medical admissions. This effect is not observed in patients undergoing emergency surgery, a primary purpose of hospitals at weekends. Third, the intensive care provided at weekends differs noticeably from that during the rest of the week in our dataset. This could be a potential source of the observed higher risk of death.

These findings should generate further research and critical evaluation of the process of providing critical care at weekends. If weekend effects were to be reproduced prospectively in comparable populations, health care providers and policy-makers alike would be obliged to take action to remove any obstacles that prevent the same quality of care being provided during the week and at weekends. Equipment, expertise, and staff need to be available in the same quantity and quality on every day of the week, especially if plannable, high-risk procedures (i.e. scheduled operations) are to be performed at weekends.

## Additional file

Additional file 1: Is supplementary material presenting Tables S1–S11 as cited in the article. (DOCX 172 kb)

## Abbreviations

CI: Confidence interval
ASDI: Austrian Centre for Documentation and Quality Assurance in Intensive Care
HR: Hazard ratio
ICU: Intensive care unit
IQR: Interquartile range
NHS: National Health Service
SAPS: Simplified Acute Physiology Score
TISS: Therapeutic Intervention Scoring System
