# Supplementary material for: Weekends affect mortality risk and chance of discharge in critically ill patients: a retrospective study in the Austrian registry for intensive care
Source: Crit Care. 2017 Sep 7;21:223. doi: 10.1186/s13054-017-1812-0 (PMC5588748; doi:10.1186/s13054-017-1812-0)
Supplement: Supplementary file 1 — Is supplementary material presenting Tables S1–S11 as cited in the article. (DOCX 172 kb) [file 13054_2017_1812_MOESM1_ESM.docx]

Supplementary Material

Weekends Affect Mortality Risk and Chance of Discharge in Critically Ill Patients: A Retrospective Study in the Austrian Registry for Intensive Care

Paul Zajic, Peter Bauer, Andrew Rhodes, Rui Moreno, Tobias Fellinger, Barbara Metnitz, Faidra Stavropoulou, Martin Posch and Philipp G. Metnitz

Table of Contents

[S1 Regional Distribution of ICUs Participating in the ASDI Project 2](#_Toc488261938)

[S2 Admission Diagnoses in Categories 3](#_Toc488261939)

[S3 Percentages of Patients Receiving Interventions 6](#_Toc488261940)

[S4 Model with Interactions (n=151,268) 8](#_Toc488261941)

[S5 Model for Hospital Mortality (n=147,397) 11](#_Toc488261942)

[S6 Model for Patients Admitted to ICUs with at least 99% Documented Hospital Outcome (n=113,161) 12](#_Toc488261943)

[S7 Models for SAPS 3 Tertiles 13](#_Toc488261944)

[S8 Model for Readmissions Only (n=14,252) 16](#_Toc488261945)

[S9 Models For Different Admission Types 17](#_Toc488261946)

[S10 Main Analysis with Cox Proportional Hazards Model (n=151,268) 22](#_Toc488261947)

[S11 Main Analysis without censoring after 30 days 23](#_Toc488261948)

Reference categories are presented in italics (without confidence intervals and p-Values).

HR Hazard Ratio
CI Confidence Interval
p p-Value
C Concordance
n Sample Size

# S1 Regional Distribution of ICUs Participating in the ASDI Project

|  | **Medical** | **Cardiac** | **Postoperative A** | **Postoperative B** | **Trauma** | **∑ in Region** |
| --- | --- | --- | --- | --- | --- | --- |
| Burgenland | 0 | 0 | 1 | 0 | 0 | 1 |
| Carinthia | 0 | 0 | 2 | 0 | 1 | 3 |
| Lower Austria | 2 | 2 | 10 | 7 | 0 | 21 |
| Upper Austria | 1 | 3 | 8 | 3 | 1 | 16 |
| Salzburg | 2 | 0 | 1 | 1 | 1 | 5 |
| Styria | 10 | 3 | 10 | 6 | 2 | 31 |
| Vienna | 10 | 6 | 4 | 16 | 2 | 38 |
| Other | 1 | 0 | 0 | 3 | 0 | 4 |
| ∑ in ICU Type | 26 | 14 | 36 | 36 | 7 | **119** |

# S2 Admission Diagnoses in Categories

|  | **Mon** | | **Tue** | | **Wed** | | **Thu** | | **Fri** | | **Sat** | | **Sun** | | **∑ of cat.** | |
| --- | --- | --- | --- | --- | --- | --- | --- | --- | --- | --- | --- | --- | --- | --- | --- | --- |
| **Admission diagnosis** | n | % | n | % | n | % | n | % | n | % | n | % | n | % | n | % |
| *Metabolic conditions* |  |  |  |  |  |  |  |  |  |  |  |  |  |  |  |  |
| Coma, metabolic | 25 | 0,1 | 33 | 0,1 | 36 | 0,1 | 27 | 0,1 | 33 | 0,1 | 24 | 0,2 | 23 | 0,2 | 201 | 0,1 |
| Intoxication, drugs | 327 | 1,3 | 310 | 1,2 | 300 | 1,2 | 306 | 1,2 | 273 | 1,1 | 293 | 2,2 | 287 | 2,3 | 2096 | 1,4 |
| Intoxication, other | 108 | 0,4 | 95 | 0,4 | 88 | 0,3 | 98 | 0,4 | 94 | 0,4 | 173 | 1,3 | 178 | 1,4 | 834 | 0,6 |
| Endocrinopathy | 9 | 0 | 26 | 0,1 | 22 | 0,1 | 18 | 0,1 | 11 | 0 | 9 | 0,1 | 4 | 0 | 99 | 0,1 |
| Diabetic ketoacidosis | 60 | 0,2 | 62 | 0,2 | 54 | 0,2 | 48 | 0,2 | 52 | 0,2 | 46 | 0,3 | 57 | 0,5 | 379 | 0,3 |
| Metabolic, other | 92 | 0,4 | 139 | 0,5 | 139 | 0,5 | 105 | 0,4 | 86 | 0,4 | 47 | 0,4 | 48 | 0,4 | 656 | 0,4 |
| *Respiratory conditions* |  |  |  |  |  |  |  |  |  |  |  |  |  |  |  |  |
| ALI / ARDS | 120 | 0,5 | 169 | 0,7 | 94 | 0,4 | 166 | 0,7 | 100 | 0,4 | 70 | 0,5 | 66 | 0,5 | 785 | 0,5 |
| Pneumonia | 538 | 2,2 | 505 | 2 | 488 | 1,9 | 449 | 1,8 | 490 | 2 | 379 | 2,9 | 408 | 3,2 | 3257 | 2,2 |
| Respiratory insufficiency / COPD | 608 | 2,5 | 600 | 2,3 | 654 | 2,5 | 595 | 2,4 | 662 | 2,7 | 542 | 4,1 | 520 | 4,1 | 4181 | 2,8 |
| Asthma attack | 18 | 0,1 | 12 | 0 | 33 | 0,1 | 16 | 0,1 | 15 | 0,1 | 21 | 0,2 | 19 | 0,2 | 134 | 0,1 |
| Pulmonary embolism | 136 | 0,6 | 144 | 0,6 | 115 | 0,4 | 126 | 0,5 | 132 | 0,5 | 95 | 0,7 | 79 | 0,6 | 827 | 0,5 |
| Foreign body airway obstruction | 8 | 0 | 14 | 0,1 | 13 | 0,1 | 11 | 0 | 8 | 0 | 7 | 0,1 | 8 | 0,1 | 69 | 0 |
| Pneumothorax | 59 | 0,2 | 51 | 0,2 | 51 | 0,2 | 56 | 0,2 | 49 | 0,2 | 33 | 0,2 | 21 | 0,2 | 320 | 0,2 |
| Pleural effusion | 30 | 0,1 | 23 | 0,1 | 22 | 0,1 | 23 | 0,1 | 34 | 0,1 | 11 | 0,1 | 15 | 0,1 | 158 | 0,1 |
| Haemoptysis | 7 | 0 | 20 | 0,1 | 21 | 0,1 | 6 | 0 | 16 | 0,1 | 4 | 0 | 12 | 0,1 | 86 | 0,1 |
| Neoplasm | 10 | 0 | 17 | 0,1 | 7 | 0 | 12 | 0 | 12 | 0 | 3 | 0 | 9 | 0,1 | 70 | 0 |
| Pulmonary oedema, non-cardiac | 36 | 0,1 | 31 | 0,1 | 27 | 0,1 | 29 | 0,1 | 36 | 0,1 | 28 | 0,2 | 22 | 0,2 | 209 | 0,1 |
| Aspiration | 88 | 0,4 | 73 | 0,3 | 84 | 0,3 | 68 | 0,3 | 88 | 0,4 | 63 | 0,5 | 67 | 0,5 | 531 | 0,4 |
| Respiratory, other | 234 | 1 | 275 | 1,1 | 282 | 1,1 | 272 | 1,1 | 245 | 1 | 173 | 1,3 | 128 | 1 | 1609 | 1,1 |
| *Cardiovascular conditions* |  |  |  |  |  |  |  |  |  |  |  |  |  |  |  |  |
| Asystole / Ventricular fibrillation, CPR | 608 | 2,5 | 578 | 2,2 | 630 | 2,4 | 567 | 2,2 | 566 | 2,4 | 551 | 4,1 | 500 | 4 | 4000 | 2,6 |
| Myocardial infarction | 748 | 3,1 | 695 | 2,7 | 957 | 3,7 | 810 | 3,2 | 716 | 3 | 509 | 3,8 | 432 | 3,4 | 4867 | 3,2 |
| Myocarditis / Cardiomyopathy | 23 | 0,1 | 30 | 0,1 | 43 | 0,2 | 38 | 0,2 | 21 | 0,1 | 16 | 0,1 | 19 | 0,2 | 190 | 0,1 |
| Unstable angina | 209 | 0,9 | 217 | 0,8 | 239 | 0,9 | 239 | 0,9 | 205 | 0,9 | 125 | 0,9 | 105 | 0,8 | 1339 | 0,9 |
| Heart failure / decompensation | 405 | 1,7 | 440 | 1,7 | 414 | 1,6 | 431 | 1,7 | 397 | 1,6 | 263 | 2 | 257 | 2 | 2607 | 1,7 |
| Pericardial effusion | 34 | 0,1 | 32 | 0,1 | 37 | 0,1 | 39 | 0,2 | 39 | 0,2 | 12 | 0,1 | 6 | 0 | 199 | 0,1 |
| Pulmonary oedema, cardiac | 199 | 0,8 | 200 | 0,8 | 165 | 0,6 | 183 | 0,7 | 170 | 0,7 | 144 | 1,1 | 181 | 1,4 | 1242 | 0,8 |
| Hypertensive crisis | 76 | 0,3 | 72 | 0,3 | 74 | 0,3 | 63 | 0,2 | 77 | 0,3 | 52 | 0,4 | 56 | 0,4 | 470 | 0,3 |
| Arrhythmia | 536 | 2,2 | 556 | 2,1 | 554 | 2,1 | 564 | 2,2 | 534 | 2,2 | 311 | 2,3 | 289 | 2,3 | 3344 | 2,2 |
| Cardioversion | 67 | 0,3 | 116 | 0,4 | 98 | 0,4 | 81 | 0,3 | 64 | 0,3 | 33 | 0,2 | 19 | 0,2 | 478 | 0,3 |
| Aortic aneurysm | 32 | 0,1 | 32 | 0,1 | 28 | 0,1 | 30 | 0,1 | 36 | 0,1 | 21 | 0,2 | 21 | 0,2 | 200 | 0,1 |
| Cardiac, other | 262 | 1,1 | 335 | 1,3 | 239 | 0,9 | 229 | 0,9 | 195 | 0,8 | 107 | 0,8 | 91 | 0,7 | 1458 | 1 |
| *Shock* |  |  |  |  |  |  |  |  |  |  |  |  |  |  |  |  |
| Shock, hypovolemic | 114 | 0,5 | 109 | 0,4 | 126 | 0,5 | 113 | 0,4 | 117 | 0,5 | 90 | 0,7 | 72 | 0,6 | 741 | 0,5 |
| Shock, septic | 133 | 0,5 | 183 | 0,7 | 141 | 0,5 | 152 | 0,6 | 147 | 0,6 | 144 | 1,1 | 125 | 1 | 1025 | 0,7 |
| Shock, cardiogenic | 113 | 0,5 | 113 | 0,4 | 100 | 0,4 | 103 | 0,4 | 90 | 0,4 | 76 | 0,6 | 74 | 0,6 | 669 | 0,4 |
| Shock, anaphylactic | 35 | 0,1 | 36 | 0,1 | 36 | 0,1 | 22 | 0,1 | 38 | 0,2 | 25 | 0,2 | 32 | 0,3 | 224 | 0,1 |
| Shock, mixed | 27 | 0,1 | 46 | 0,2 | 23 | 0,1 | 42 | 0,2 | 32 | 0,1 | 15 | 0,1 | 27 | 0,2 | 212 | 0,1 |
| Shock, unknown origin | 27 | 0,1 | 27 | 0,1 | 39 | 0,2 | 24 | 0,1 | 27 | 0,1 | 18 | 0,1 | 16 | 0,1 | 178 | 0,1 |
| *Renal conditions* |  |  |  |  |  |  |  |  |  |  |  |  |  |  |  |  |
| Acute renal failure | 336 | 1,4 | 299 | 1,2 | 285 | 1,1 | 337 | 1,3 | 356 | 1,5 | 270 | 2 | 265 | 2,1 | 2148 | 1,4 |
| Chronic renal failure | 91 | 0,4 | 83 | 0,3 | 97 | 0,4 | 97 | 0,4 | 93 | 0,4 | 68 | 0,5 | 62 | 0,5 | 591 | 0,4 |
| Crush syndrome | 12 | 0 | 5 | 0 | 4 | 0 | 3 | 0 | 6 | 0 | 4 | 0 | 7 | 0,1 | 41 | 0 |
| Renal, other | 9 | 0 | 11 | 0 | 2 | 0 | 12 | 0 | 10 | 0 | 4 | 0 | 4 | 0 | 52 | 0 |
| *Neurologic conditions* |  |  |  |  |  |  |  |  |  |  |  |  |  |  |  |  |
| Cerebrovascular insult | 201 | 0,8 | 183 | 0,7 | 213 | 0,8 | 195 | 0,8 | 216 | 0,9 | 170 | 1,3 | 184 | 1,5 | 1362 | 0,9 |
| Intracranial mass lesion | 147 | 0,6 | 114 | 0,4 | 126 | 0,5 | 111 | 0,4 | 141 | 0,6 | 105 | 0,8 | 112 | 0,9 | 856 | 0,6 |
| Meningitis / encephalitis | 22 | 0,1 | 34 | 0,1 | 27 | 0,1 | 33 | 0,1 | 44 | 0,2 | 35 | 0,3 | 23 | 0,2 | 218 | 0,1 |
| Seizure | 171 | 0,7 | 146 | 0,6 | 156 | 0,6 | 183 | 0,7 | 167 | 0,7 | 152 | 1,1 | 141 | 1,1 | 1116 | 0,7 |
| Myopathy / myasthenia | 14 | 0,1 | 17 | 0,1 | 17 | 0,1 | 19 | 0,1 | 14 | 0,1 | 5 | 0 | 12 | 0,1 | 98 | 0,1 |
| Degenerative disease | 7 | 0 | 8 | 0 | 7 | 0 | 10 | 0 | 8 | 0 | 8 | 0,1 | 5 | 0 | 53 | 0 |
| Coma, stupor | 183 | 0,8 | 211 | 0,8 | 172 | 0,7 | 201 | 0,8 | 177 | 0,7 | 158 | 1,2 | 185 | 1,5 | 1287 | 0,9 |
| Focal neurologic deficit | 20 | 0,1 | 27 | 0,1 | 18 | 0,1 | 21 | 0,1 | 20 | 0,1 | 23 | 0,2 | 26 | 0,2 | 155 | 0,1 |
| Neurologic, other | 142 | 0,6 | 155 | 0,6 | 127 | 0,5 | 155 | 0,6 | 152 | 0,6 | 102 | 0,8 | 109 | 0,9 | 942 | 0,6 |
| *Sepsis* |  |  |  |  |  |  |  |  |  |  |  |  |  |  |  |  |
| Sepsis, suspected | 145 | 0,6 | 172 | 0,7 | 180 | 0,7 | 160 | 0,6 | 173 | 0,7 | 132 | 1 | 123 | 1 | 1085 | 0,7 |
| Sepsis, documented | 103 | 0,4 | 124 | 0,5 | 115 | 0,4 | 125 | 0,5 | 140 | 0,6 | 77 | 0,6 | 75 | 0,6 | 759 | 0,5 |
| Sepsis, undocumented | 14 | 0,1 | 20 | 0,1 | 13 | 0,1 | 21 | 0,1 | 13 | 0,1 | 11 | 0,1 | 9 | 0,1 | 101 | 0,1 |
| Sepsis, unknown | 44 | 0,2 | 35 | 0,1 | 48 | 0,2 | 57 | 0,2 | 45 | 0,2 | 32 | 0,2 | 36 | 0,3 | 297 | 0,2 |
| *Trauma, non-operated* |  |  |  |  |  |  |  |  |  |  |  |  |  |  |  |  |
| Traumatic brain injury / spinal trauma | 220 | 0,9 | 217 | 0,8 | 259 | 1 | 253 | 1 | 242 | 1 | 252 | 1,9 | 201 | 1,6 | 1644 | 1,1 |
| Thoracic trauma | 68 | 0,3 | 77 | 0,3 | 80 | 0,3 | 60 | 0,2 | 86 | 0,4 | 98 | 0,7 | 64 | 0,5 | 533 | 0,4 |
| Abdominal trauma | 23 | 0,1 | 15 | 0,1 | 17 | 0,1 | 6 | 0 | 15 | 0,1 | 19 | 0,1 | 16 | 0,1 | 111 | 0,1 |
| Skeletal trauma | 86 | 0,4 | 88 | 0,3 | 87 | 0,3 | 89 | 0,4 | 75 | 0,3 | 68 | 0,5 | 56 | 0,4 | 549 | 0,4 |
| Polytrauma | 67 | 0,3 | 56 | 0,2 | 52 | 0,2 | 68 | 0,3 | 61 | 0,3 | 52 | 0,4 | 48 | 0,4 | 404 | 0,3 |
| Burns | 23 | 0,1 | 18 | 0,1 | 16 | 0,1 | 18 | 0,1 | 8 | 0 | 15 | 0,1 | 20 | 0,2 | 118 | 0,1 |
| Trauma, other | 55 | 0,2 | 60 | 0,2 | 70 | 0,3 | 67 | 0,3 | 69 | 0,3 | 49 | 0,4 | 55 | 0,4 | 425 | 0,3 |
| *Gastrointestinal conditions* |  |  |  |  |  |  |  |  |  |  |  |  |  |  |  |  |
| Liver failure | 48 | 0,2 | 40 | 0,2 | 53 | 0,2 | 51 | 0,2 | 48 | 0,2 | 23 | 0,2 | 29 | 0,2 | 292 | 0,2 |
| Pancreatitis | 74 | 0,3 | 75 | 0,3 | 77 | 0,3 | 73 | 0,3 | 86 | 0,4 | 68 | 0,5 | 59 | 0,5 | 512 | 0,3 |
| Upper GI bleed | 180 | 0,7 | 174 | 0,7 | 193 | 0,7 | 189 | 0,7 | 186 | 0,8 | 153 | 1,2 | 155 | 1,2 | 1230 | 0,8 |
| Lower GI bleed | 43 | 0,2 | 39 | 0,2 | 43 | 0,2 | 40 | 0,2 | 39 | 0,2 | 32 | 0,2 | 39 | 0,3 | 275 | 0,2 |
| Acute abdomen | 58 | 0,2 | 68 | 0,3 | 62 | 0,2 | 52 | 0,2 | 57 | 0,2 | 49 | 0,4 | 33 | 0,3 | 379 | 0,3 |
| Gastrointestinal, other | 48 | 0,2 | 69 | 0,3 | 63 | 0,2 | 57 | 0,2 | 61 | 0,3 | 22 | 0,2 | 35 | 0,3 | 355 | 0,2 |
| *Haematological conditions* |  |  |  |  |  |  |  |  |  |  |  |  |  |  |  |  |
| Bone marrow transplant | 1 | 0 | 1 | 0 | 0 | 0 | 1 | 0 | 0 | 0 | 1 | 0 | 1 | 0 | 5 | 0 |
| DIC | 1 | 0 | 5 | 0 | 3 | 0 | 1 | 0 | 4 | 0 | 3 | 0 | 1 | 0 | 18 | 0 |
| Malign haematological disease | 37 | 0,2 | 37 | 0,1 | 32 | 0,1 | 33 | 0,1 | 31 | 0,1 | 9 | 0,1 | 6 | 0 | 185 | 0,1 |
| Non-malign haematological disease | 18 | 0,1 | 14 | 0,1 | 17 | 0,1 | 17 | 0,1 | 17 | 0,1 | 9 | 0,1 | 2 | 0 | 94 | 0,1 |
| *Other conditions* |  |  |  |  |  |  |  |  |  |  |  |  |  |  |  |  |
| Other | 284 | 1,2 | 317 | 1,2 | 322 | 1,2 | 339 | 1,3 | 314 | 1,3 | 241 | 1,8 | 252 | 2 | 2069 | 1,4 |
| *Obstetric conditions* |  |  |  |  |  |  |  |  |  |  |  |  |  |  |  |  |
| Preeclampsia / eclampsia | 4 | 0 | 17 | 0,1 | 9 | 0 | 7 | 0 | 10 | 0 | 4 | 0 | 6 | 0 | 57 | 0 |
| HELLP syndrome | 12 | 0 | 13 | 0,1 | 4 | 0 | 9 | 0 | 5 | 0 | 4 | 0 | 5 | 0 | 52 | 0 |
| Obstetric, other | 8 | 0 | 9 | 0 | 6 | 0 | 8 | 0 | 8 | 0 | 1 | 0 | 4 | 0 | 44 | 0 |
| *Thoracic surgery* |  |  |  |  |  |  |  |  |  |  |  |  |  |  |  |  |
| Pneumonectomy | 33 | 0,1 | 22 | 0,1 | 20 | 0,1 | 22 | 0,1 | 29 | 0,1 | 3 | 0 | 2 | 0 | 131 | 0,1 |
| Lobectomy | 104 | 0,4 | 74 | 0,3 | 74 | 0,3 | 85 | 0,3 | 86 | 0,4 | 2 | 0 | 6 | 0 | 431 | 0,3 |
| Pleurectomy / pleural surgery | 34 | 0,1 | 42 | 0,2 | 41 | 0,2 | 52 | 0,2 | 58 | 0,2 | 12 | 0,1 | 15 | 0,1 | 254 | 0,2 |
| Pneumothorax | 13 | 0,1 | 23 | 0,1 | 28 | 0,1 | 12 | 0 | 24 | 0,1 | 16 | 0,1 | 16 | 0,1 | 132 | 0,1 |
| Thoracic, other | 116 | 0,5 | 114 | 0,4 | 165 | 0,6 | 118 | 0,5 | 133 | 0,6 | 31 | 0,2 | 37 | 0,3 | 714 | 0,5 |
| *Cardiovascular surgery* |  |  |  |  |  |  |  |  |  |  |  |  |  |  |  |  |
| Valvular surgery | 478 | 2 | 436 | 1,7 | 428 | 1,7 | 402 | 1,6 | 323 | 1,3 | 90 | 0,7 | 158 | 1,3 | 2315 | 1,5 |
| CABG | 437 | 1,8 | 354 | 1,4 | 341 | 1,3 | 389 | 1,5 | 375 | 1,6 | 165 | 1,2 | 160 | 1,3 | 2221 | 1,5 |
| Carotid surgery | 220 | 0,9 | 249 | 1 | 236 | 0,9 | 259 | 1 | 286 | 1,2 | 21 | 0,2 | 20 | 0,2 | 1291 | 0,9 |
| Valvular surgery + CABG | 148 | 0,6 | 157 | 0,6 | 120 | 0,5 | 126 | 0,5 | 150 | 0,6 | 42 | 0,3 | 48 | 0,4 | 791 | 0,5 |
| Abdominal aortal surgery | 229 | 0,9 | 259 | 1 | 244 | 0,9 | 230 | 0,9 | 210 | 0,9 | 36 | 0,3 | 23 | 0,2 | 1231 | 0,8 |
| Thoracic aortal surgery | 84 | 0,3 | 106 | 0,4 | 94 | 0,4 | 88 | 0,3 | 90 | 0,4 | 39 | 0,3 | 32 | 0,3 | 533 | 0,4 |
| Peripheral vascular surgery | 403 | 1,7 | 423 | 1,6 | 360 | 1,4 | 352 | 1,4 | 410 | 1,7 | 76 | 0,6 | 71 | 0,6 | 2095 | 1,4 |
| Cardiovascular, other | 102 | 0,4 | 154 | 0,6 | 138 | 0,5 | 133 | 0,5 | 121 | 0,5 | 45 | 0,3 | 37 | 0,3 | 730 | 0,5 |
| *Neurosurgery* |  |  |  |  |  |  |  |  |  |  |  |  |  |  |  |  |
| Intracranial tumour | 637 | 2,6 | 621 | 2,4 | 646 | 2,5 | 668 | 2,6 | 594 | 2,5 | 32 | 0,2 | 41 | 0,3 | 3239 | 2,1 |
| Intracranial bleed | 479 | 2 | 477 | 1,8 | 523 | 2 | 514 | 2 | 459 | 1,9 | 371 | 2,8 | 346 | 2,8 | 3169 | 2,1 |
| Spinal surgery | 95 | 0,4 | 118 | 0,5 | 88 | 0,3 | 104 | 0,4 | 107 | 0,4 | 25 | 0,2 | 20 | 0,2 | 557 | 0,4 |
| Neurosurgical, other | 203 | 0,8 | 303 | 1,2 | 262 | 1 | 308 | 1,2 | 197 | 0,8 | 72 | 0,5 | 57 | 0,5 | 1402 | 0,9 |
| *Transplant surgery* |  |  |  |  |  |  |  |  |  |  |  |  |  |  |  |  |
| Liver | 14 | 0,1 | 18 | 0,1 | 15 | 0,1 | 23 | 0,1 | 16 | 0,1 | 19 | 0,1 | 21 | 0,2 | 126 | 0,1 |
| Heart | 8 | 0 | 12 | 0 | 15 | 0,1 | 12 | 0 | 11 | 0 | 8 | 0,1 | 7 | 0,1 | 73 | 0 |
| Kidneys | 25 | 0,1 | 14 | 0,1 | 15 | 0,1 | 10 | 0 | 23 | 0,1 | 20 | 0,2 | 7 | 0,1 | 114 | 0,1 |
| Pancreas | 0 | 0 | 0 | 0 | 2 | 0 | 0 | 0 | 0 | 0 | 1 | 0 | 0 | 0 | 3 | 0 |
| Lungs | 38 | 0,2 | 43 | 0,2 | 32 | 0,1 | 26 | 0,1 | 41 | 0,2 | 39 | 0,3 | 33 | 0,3 | 252 | 0,2 |
| Other | 0 | 0 | 4 | 0 | 2 | 0 | 1 | 0 | 3 | 0 | 1 | 0 | 1 | 0 | 12 | 0 |
| *Trauma, operated* |  |  |  |  |  |  |  |  |  |  |  |  |  |  |  |  |
| Polytrauma | 193 | 0,8 | 194 | 0,8 | 206 | 0,8 | 217 | 0,9 | 230 | 1 | 264 | 2 | 181 | 1,4 | 1485 | 1 |
| Traumatic brain injury / spinal trauma | 415 | 1,7 | 353 | 1,4 | 372 | 1,4 | 396 | 1,6 | 425 | 1,8 | 381 | 2,9 | 313 | 2,5 | 2655 | 1,8 |
| Abdominal trauma | 43 | 0,2 | 61 | 0,2 | 45 | 0,2 | 42 | 0,2 | 50 | 0,2 | 52 | 0,4 | 59 | 0,5 | 352 | 0,2 |
| Thoracic trauma | 72 | 0,3 | 109 | 0,4 | 97 | 0,4 | 108 | 0,4 | 100 | 0,4 | 98 | 0,7 | 64 | 0,5 | 648 | 0,4 |
| Other | 713 | 2,9 | 691 | 2,7 | 672 | 2,6 | 729 | 2,9 | 755 | 3,1 | 462 | 3,5 | 433 | 3,4 | 4455 | 2,9 |
| *Gastrointestinal surgery* |  |  |  |  |  |  |  |  |  |  |  |  |  |  |  |  |
| Biliary tract | 140 | 0,6 | 193 | 0,7 | 213 | 0,8 | 188 | 0,7 | 177 | 0,7 | 62 | 0,5 | 58 | 0,5 | 1031 | 0,7 |
| Liver | 140 | 0,6 | 182 | 0,7 | 143 | 0,6 | 136 | 0,5 | 139 | 0,6 | 13 | 0,1 | 10 | 0,1 | 763 | 0,5 |
| Pancreas | 192 | 0,8 | 189 | 0,7 | 215 | 0,8 | 198 | 0,8 | 213 | 0,9 | 19 | 0,1 | 16 | 0,1 | 1042 | 0,7 |
| Stomach | 281 | 1,2 | 289 | 1,1 | 309 | 1,2 | 279 | 1,1 | 177 | 0,7 | 26 | 0,2 | 28 | 0,2 | 1389 | 0,9 |
| Mesentery | 123 | 0,5 | 154 | 0,6 | 132 | 0,5 | 127 | 0,5 | 109 | 0,5 | 27 | 0,2 | 26 | 0,2 | 698 | 0,5 |
| Exploratory laparotomy | 105 | 0,4 | 100 | 0,4 | 98 | 0,4 | 116 | 0,5 | 114 | 0,5 | 61 | 0,5 | 71 | 0,6 | 665 | 0,4 |
| Gastrointestinal bleed | 115 | 0,5 | 104 | 0,4 | 113 | 0,4 | 98 | 0,4 | 96 | 0,4 | 66 | 0,5 | 79 | 0,6 | 671 | 0,4 |
| Perforation / obstruction | 748 | 3,1 | 786 | 3 | 758 | 2,9 | 798 | 3,2 | 743 | 3,1 | 502 | 3,8 | 460 | 3,7 | 4795 | 3,2 |
| Other | 392 | 1,6 | 455 | 1,8 | 484 | 1,9 | 457 | 1,8 | 388 | 1,6 | 96 | 0,7 | 74 | 0,6 | 2346 | 1,6 |
| *Other surgery* |  |  |  |  |  |  |  |  |  |  |  |  |  |  |  |  |
| ORL | 121 | 0,5 | 160 | 0,6 | 137 | 0,5 | 149 | 0,6 | 121 | 0,5 | 42 | 0,3 | 36 | 0,3 | 766 | 0,5 |
| Urology | 389 | 1,6 | 384 | 1,5 | 407 | 1,6 | 385 | 1,5 | 313 | 1,3 | 39 | 0,3 | 27 | 0,2 | 1944 | 1,3 |
| Orthopaedics | 686 | 2,8 | 875 | 3,4 | 871 | 3,4 | 785 | 3,1 | 704 | 2,9 | 36 | 0,3 | 39 | 0,3 | 3996 | 2,6 |
| Gynaecology | 140 | 0,6 | 183 | 0,7 | 278 | 1,1 | 212 | 0,8 | 190 | 0,8 | 57 | 0,4 | 65 | 0,5 | 1125 | 0,7 |
| Carcinoma | 616 | 2,5 | 794 | 3,1 | 800 | 3,1 | 819 | 3,2 | 583 | 2,4 | 21 | 0,2 | 24 | 0,2 | 3657 | 2,4 |
| Other | 93 | 0,4 | 107 | 0,4 | 94 | 0,4 | 93 | 0,4 | 80 | 0,3 | 36 | 0,3 | 32 | 0,3 | 535 | 0,4 |
| *Not documented* | 5570 | 22,9 | 6011 | 23,2 | 5932 | 23 | 5707 | 22,5 | 5518 | 22,9 | 2692 | 20,3 | 2548 | 20,3 | 33978 | 22,5 |
| ∑ at weekday | 24367 | | 25866 | | 25810 | | 25312 | | 24075 | | 13279 | | 12559 | | 151268 | |

# S3 Percentages of Patients Receiving Interventions

|  | **On Admission Day (stratified by Admission Day)** | | | | | | | |
| --- | --- | --- | --- | --- | --- | --- | --- | --- |
| Intervention | Mon | Tue | Wed | Thu | Fri | Sat | Sun | *p* |
| Supplementary ventilator support | 41.3% | 40.7% | 41.3% | 40.8% | 41.0% | 38.2% | 38.0% | <0.001 |
| Mechanical ventilation | 47.9% | 48.8% | 47.6% | 48.0% | 48.1% | 48.2% | 48.3% | 1.00 |
| Care of artificial airways | 39.5% | 40.1% | 39.1% | 39.3% | 39.5% | 38.1% | 36.9% | <0.001 |
| Quantitative urine output measurement | 91.4% | 91.1% | 90.4% | 90.7% | 90.6% | 88.4% | 88.4% | <0.001 |
| Laboratory | 93.7% | 93.5% | 93.1% | 93.5% | 92.9% | 92.4% | 92.7% | <0.001 |
| Measurement of intracranial pressure | 1.5% | 1.4% | 1.3% | 1.5% | 1.5% | 2.6% | 2.4% | <0.001 |
| Enteral feeding | 12.5% | 13.1% | 13.0% | 12.2% | 13.3% | 12.3% | 12.2% | 0.01 |
| Intravenous hyperalimentation | 16.2% | 17.3% | 17.0% | 17.0% | 18.0% | 14.1% | 13.8% | <0.001 |
| Hemofiltration techniques | 2.6% | 2.4% | 2.4% | 2.3% | 2.7% | 3.6% | 3.6% | <0.001 |
| Treatment for improving lung function | 76.9% | 76.9% | 76.5% | 75.6% | 76.0% | 71.6% | 72.0% | <0.001 |
| Routine dressing changes | 83.6% | 84.3% | 84.1% | 83.7% | 82.9% | 78.9% | 78.6% | <0.001 |
| Frequent dressing changes | 25.4% | 26.2% | 25.4% | 25.8% | 25.2% | 21.7% | 20.8% | <0.001 |
| Treatment of metabolic acidosis/alkalosis | 6.3% | 6.3% | 5.8% | 6.4% | 6.7% | 9.8% | 10.4% | <0.001 |
| Active diuresis | 16.8% | 17.1% | 16.4% | 16.9% | 17.0% | 17.8% | 17.6% | 0.23 |
| Intravenous replacement of large fluid losses | 22.0% | 21.3% | 21.4% | 22.2% | 21.7% | 21.9% | 21.5% | 1.00 |
| Single vasoactive medication | 31.2% | 31.7% | 31.1% | 30.8% | 31.6% | 31.5% | 31.9% | 1.00 |
| Multiple vasoactive medication | 12.4% | 12.8% | 11.8% | 12.5% | 12.9% | 13.7% | 13.6% | <0.001 |
| Single medication | 5.4% | 5.5% | 5.5% | 5.7% | 5.5% | 5.7% | 5.4% | 1.00 |
| Multiple intravenous medication | 89.7% | 89.5% | 89.1% | 88.9% | 88.7% | 87.1% | 87.6% | <0.001 |
| Single specific intervention in the ICU | 13.8% | 13.7% | 14.3% | 13.6% | 13.8% | 16.0% | 16.2% | <0.001 |
| Multiple specific interventions in the ICU | 10.2% | 10.6% | 10.4% | 10.2% | 11.1% | 12.3% | 12.2% | <0.001 |
| Standard monitoring | 97.9% | 97.9% | 97.7% | 97.7% | 97.5% | 96.8% | 96.9% | <0.001 |
| Cardiopulmonary resuscitation | 2.8% | 2.5% | 2.5% | 2.6% | 2.6% | 4.0% | 3.9% | <0.001 |
| Specific interventions outside the ICU | 22.2% | 21.9% | 22.2% | 23.0% | 22.6% | 26.9% | 26.9% | <0.001 |
| Central venous line | 54.1% | 53.7% | 52.6% | 53.1% | 54.0% | 49.6% | 49.2% | <0.001 |
| Peripheral arterial line | 75.7% | 76.1% | 75.4% | 75.8% | 75.5% | 70.1% | 69.4% | <0.001 |
| Left atrium monitoring | 1.7% | 1.9% | 1.9% | 1.8% | 1.8% | 1.6% | 1.5% | 1.00 |
| Care of drains | 45.5% | 46.8% | 45.7% | 46.0% | 46.1% | 32.6% | 30.4% | <0.001 |

|  | **During ICU Stay (stratified by Day of the Week)** | | | | | | | | | | | | | | |  |
| --- | --- | --- | --- | --- | --- | --- | --- | --- | --- | --- | --- | --- | --- | --- | --- | --- |
| Intervention | Mon | Tue | | Wed | | Thu | | Fri | | Sat | | Sun | | *p* | |  |
| Supplementary ventilator support | 33.8% | 34.6% | | 35.1% | | 35.2% | | 35.2% | | 34.7% | | 33.5% | | <0.001 | |  |
| Mechanical ventilation | 58.0% | 57.2% | | 56.9% | | 56.6% | | 56.6% | | 57.6% | | 58.6% | | <0.001 | |  |
| Care of artificial airways | 48.2% | 47.5% | | 47.0% | | 46.7% | | 46.5% | | 47.1% | | 48.1% | | <0.001 | |  |
| Quantitative urine output measurement | 95.5% | 95.6% | | 95.5% | | 95.4% | | 95.3% | | 95.5% | | 95.6% | | 0.28 | |  |
| Laboratory | 95.4% | 95.2% | | 95.3% | | 95.2% | | 95.2% | | 95.1% | | 94.8% | | <0.001 | |  |
| Measurement of intracranial pressure | 4.3% | 4.1% | | 4.0% | | 4.0% | | 4.0% | | 4.0% | | 4.2% | | 0.00 | |  |
| Enteral feeding | 51.7% | 49.7% | | 49.1% | | 48.9% | | 48.7% | | 49.4% | | 51.1% | | <0.001 | |  |
| Intravenous hyperalimentation | 40.6% | 39.6% | | 39.6% | | 39.8% | | 39.8% | | 40.6% | | 41.0% | | <0.001 | |  |
| Hemofiltration techniques | 9.8% | 9.3% | | 9.4% | | 9.2% | | 9.3% | | 9.5% | | 9.6% | | <0.001 | |  |
| Treatment for improving lung function | 86.6% | 86.5% | | 86.6% | | 86.6% | | 86.3% | | 86.5% | | 86.2% | | 0.99 | |  |
| Routine dressing changes | 90.7% | 90.7% | | 90.9% | | 90.9% | | 90.8% | | 90.8% | | 90.8% | | 1.00 | |  |
| Frequent dressing changes | 28.5% | 28.5% | | 28.8% | | 29.0% | | 29.1% | | 29.0% | | 28.9% | | 0.39 | |  |
| Treatment of metabolic acidosis/alkalosis | 3.5% | 3.4% | | 3.4% | | 3.4% | | 3.4% | | 3.5% | | 3.7% | | 0.15 | |  |
| Active diuresis | 37.5% | 36.1% | | 35.7% | | 35.6% | | 35.4% | | 36.3% | | 37.8% | | <0.001 | |  |
| Intravenous replacement of large fluid losses | 8.9% | 9.5% | | 9.6% | | 9.7% | | 9.8% | | 10.0% | | 9.5% | | <0.001 | |  |
| Single vasoactive medication | 31.7% | 31.5% | | 31.6% | | 31.6% | | 31.8% | | 32.6% | | 32.4% | | <0.001 | |  |
| Multiple vasoactive medication | 14.3% | 14.2% | | 14.4% | | 14.4% | | 14.4% | | 14.6% | | 14.6% | | 1.00 | |  |
| Single medication | 2.3% | 2.5% | | 2.5% | | 2.5% | | 2.6% | | 2.5% | | 2.4% | | 0.00 | |  |
| Multiple intravenous medication | 94.9% | 94.7% | | 94.8% | | 94.7% | | 94.5% | | 94.7% | | 94.9% | | 0.00 | |  |
| Single specific intervention in the ICU | 11.2% | 11.2% | | 11.0% | | 11.0% | | 11.4% | | 9.2% | | 9.0% | | <0.001 | |  |
| Multiple specific interventions in the ICU | 7.8% | 7.8% | | 7.7% | | 7.7% | | 7.8% | | 7.1% | | 7.2% | | <0.001 | |  |
| Standard monitoring | 98.0% | 98.1% | | 98.1% | | 98.0% | | 97.9% | | 98.0% | | 98.0% | | 1.00 | |  |
| Cardiopulmonary resuscitation | 0.7% | 0.7% | | 0.7% | | 0.7% | | 0.7% | | 0.6% | | 0.7% | | 1.00 | |  |
| Specific interventions outside the ICU | 14.3% | 13.8% | | 13.8% | | 13.4% | | 14.3% | | 9.5% | | 8.6% | | <0.001 | |  |
| Central venous line | 79.9% | 78.4% | | 78.1% | | 78.0% | | 78.1% | | 78.7% | | 79.9% | | <0.001 | |  |
| Peripheral arterial line | 82.4% | 82.8% | | 83.1% | | 83.5% | | 83.4% | | 83.7% | | 83.1% | | <0.001 | |  |
| Left atrium monitoring | 1.9% | 1.9% | | 1.9% | | 2.0% | | 2.0% | | 2.1% | | 2.0% | | 0.15 | |  |
| Care of drains | 47.9% | | 48.7% | | 49.7% | | 50.4% | | 50.8% | | 51.0% | | 49.3% | | <0.001 | |

# S4 Model with Interactions (n=151,268)

|  | Death in ICU (C=0.864) | | |  | ICU Discharge (C=0.730) | | |
| --- | --- | --- | --- | --- | --- | --- | --- |
|  | HR | 95% CI | p |  | HR | 95% CI | p |
| SAPS 3 [per 10 points] | 1.848 | (1.802-1.895) | 0.000 |  | 0.656 | (0.649-0.663) | 0.000 |
| Year of admission |  |  |  |  |  |  |  |
| *2012* | 1.000 |  |  |  | 1.000 |  |  |
| 2013 | 1.019 | (0.971-1.069) | 0.450 |  | 0.997 | (0.983-1.012) | 0.729 |
| 2014 | 0.988 | (0.937-1.042) | 0.665 |  | 1.048 | (1.031-1.066) | 0.000 |
| 2015 | 0.928 | (0.880-0.978) | 0.006 |  | 1.087 | (1.069-1.106) | 0.000 |
| Month of admission |  |  |  |  |  |  |  |
| *January* | 1.000 |  |  |  | 1.000 |  |  |
| February | 1.051 | (0.970-1.140) | 0.224 |  | 0.979 | (0.954-1.005) | 0.110 |
| March | 1.012 | (0.933-1.096) | 0.780 |  | 0.997 | (0.972-1.023) | 0.836 |
| April | 0.970 | (0.893-1.054) | 0.471 |  | 0.996 | (0.971-1.023) | 0.782 |
| May | 1.008 | (0.927-1.095) | 0.856 |  | 1.007 | (0.981-1.033) | 0.623 |
| June | 1.017 | (0.937-1.105) | 0.684 |  | 1.006 | (0.980-1.032) | 0.654 |
| July | 0.959 | (0.882-1.042) | 0.323 |  | 1.011 | (0.985-1.037) | 0.403 |
| August | 1.079 | (0.994-1.171) | 0.070 |  | 0.985 | (0.959-1.011) | 0.254 |
| September | 1.018 | (0.938-1.106) | 0.668 |  | 1.000 | (0.974-1.026) | 0.990 |
| October | 0.963 | (0.887-1.046) | 0.377 |  | 0.999 | (0.974-1.025) | 0.943 |
| November | 0.995 | (0.916-1.082) | 0.913 |  | 0.994 | (0.969-1.020) | 0.666 |
| December | 0.992 | (0.914-1.078) | 0.857 |  | 0.987 | (0.962-1.013) | 0.334 |
| Admission type |  |  |  |  |  |  |  |
| *medical* | 1.000 |  |  |  | 1.000 |  |  |
| unscheduled surgery | 0.656 | (0.573-0.750) | 0.000 |  | 0.891 | (0.853-0.931) | 0.000 |
| scheduled surgery | 0.322 | (0.270-0.385) | 0.000 |  | 1.277 | (1.233-1.323) | 0.000 |
| surgery unspecified | 0.930 | (0.767-1.126) | 0.455 |  | 0.835 | (0.791-0.882) | 0.000 |
| unknown | 1.173 | (0.869-1.583) | 0.298 |  | 1.049 | (0.974-1.128) | 0.205 |
| Weekday of admission |  |  |  |  |  |  |  |
| Monday | 1.039 | (0.925-1.167) | 0.521 |  | 0.905 | (0.875-0.937) | 0.000 |
| Tuesday | 1.039 | (0.926-1.165) | 0.517 |  | 0.969 | (0.937-1.003) | 0.070 |
| *Wednesday* | 1.000 |  |  |  | 1.000 |  |  |
| Thursday | 0.949 | (0.842-1.069) | 0.385 |  | 1.014 | (0.980-1.048) | 0.429 |
| Friday | 1.091 | (0.970-1.226) | 0.145 |  | 1.039 | (1.003-1.075) | 0.031 |
| Saturday | 1.206 | (1.068-1.363) | 0.003 |  | 1.021 | (0.984-1.059) | 0.274 |
| Sunday | 1.184 | (1.047-1.339) | 0.007 |  | 0.947 | (0.913-0.983) | 0.004 |
| Weekday of event |  |  |  |  |  |  |  |
| Monday | 0.992 | (0.929-1.059) | 0.805 |  | 1.006 | (0.985-1.027) | 0.606 |
| Tuesday | 1.027 | (0.964-1.094) | 0.404 |  | 1.016 | (0.996-1.037) | 0.108 |
| *Wednesday* | 1.000 |  |  |  |  |  |  |
| Thursday | 1.027 | (0.964-1.095) | 0.404 |  | 0.972 | (0.953-0.991) | 0.004 |
| Friday | 1.010 | (0.947-1.077) | 0.765 |  | 0.996 | (0.977-1.017) | 0.729 |
| Saturday | 0.937 | (0.877-1.001) | 0.053 |  | 0.627 | (0.613-0.642) | 0.000 |
| Sunday | 0.858 | (0.801-0.918) | 0.000 |  | 0.556 | (0.542-0.570) | 0.000 |
| Interaction: Admission type x Weekday of admission | | | | | | | |
| Unscheduled surgery |  |  |  |  |  |  |  |
| Monday | 1.016 | (0.860-1.201) | 0.849 |  | 1.013 | (0.953-1.078) | 0.671 |
| Tuesday | 0.975 | (0.827-1.149) | 0.761 |  | 1.004 | (0.944-1.067) | 0.901 |
| *Wednesday* |  |  |  |  |  |  |  |
| Thursday | 0.989 | (0.836-1.170) | 0.897 |  | 1.043 | (0.982-1.108) | 0.172 |
| Friday | 0.988 | (0.841-1.162) | 0.888 |  | 1.057 | (0.995-1.122) | 0.072 |
| Saturday | 0.885 | (0.743-1.054) | 0.169 |  | 1.010 | (0.948-1.077) | 0.755 |
| Sunday | 0.910 | (0.763-1.086) | 0.298 |  | 0.992 | (0.929-1.060) | 0.811 |
| Scheduled surgery |  |  |  |  |  |  |  |
| Monday | 0.998 | (0.788-1.263) | 0.985 |  | 1.099 | (1.048-1.153) | 0.000 |
| Tuesday | 0.795 | (0.619-1.020) | 0.071 |  | 1.035 | (0.987-1.085) | 0.153 |
| *Wednesday* |  |  |  |  |  |  |  |
| Thursday | 1.105 | (0.871-1.401) | 0.412 |  | 0.996 | (0.950-1.044) | 0.880 |
| Friday | 0.966 | (0.758-1.232) | 0.782 |  | 1.051 | (1.002-1.103) | 0.042 |
| Saturday | 1.561 | (1.142-2.135) | 0.005 |  | 0.801 | (0.745-0.861) | 0.000 |
| Sunday | 1.451 | (1.032-2.041) | 0.032 |  | 0.854 | (0.794-0.920) | 0.000 |
| Surgery unspecified |  |  |  |  |  |  |  |
| Monday | 1.147 | (0.913-1.441) | 0.238 |  | 1.035 | (0.968-1.107) | 0.312 |
| Tuesday | 1.012 | (0.809-1.266) | 0.917 |  | 1.027 | (0.961-1.098) | 0.427 |
| *Wednesday* |  |  |  |  |  |  |  |
| Thursday | 1.000 | (0.792-1.262) | 0.997 |  | 1.041 | (0.974-1.113) | 0.236 |
| Friday | 0.929 | (0.742-1.163) | 0.522 |  | 1.042 | (0.975-1.114) | 0.226 |
| Saturday | 1.174 | (0.931-1.482) | 0.175 |  | 0.977 | (0.905-1.054) | 0.543 |
| Sunday | 0.960 | (0.757-1.217) | 0.737 |  | 0.971 | (0.899-1.049) | 0.455 |
| Unknown |  |  |  |  |  |  |  |
| Monday | 1.145 | (0.779-1.683) | 0.490 |  | 1.034 | (0.941-1.137) | 0.485 |
| Tuesday | 0.874 | (0.582-1.312) | 0.515 |  | 0.956 | (0.871-1.051) | 0.352 |
| *Wednesday* |  |  |  |  |  |  |  |
| Thursday | 0.938 | (0.618-1.422) | 0.763 |  | 0.917 | (0.835-1.007) | 0.069 |
| Friday | 1.283 | (0.867-1.899) | 0.213 |  | 0.921 | (0.838-1.011) | 0.084 |
| Saturday | 1.078 | (0.710-1.635) | 0.726 |  | 0.949 | (0.853-1.055) | 0.330 |
| Sunday | 1.045 | (0.681-1.604) | 0.839 |  | 0.928 | (0.833-1.034) | 0.177 |

| Interaction: SAPS 3 [per 10 points] x Admission type | | | | | | | |
| --- | --- | --- | --- | --- | --- | --- | --- |
| *medical* |  |  |  |  |  |  |  |
| unscheduled surgery | 1.065 | (1.038-1.093) | 0.000 |  | 1.094 | (1.082-1.106) | 0.000 |
| scheduled surgery | 1.364 | (1.308-1.423) | 0.000 |  | 1.145 | (1.133-1.157) | 0.000 |
| surgery unspecified | 1.250 | (1.206-1.294) | 0.000 |  | 0.965 | (0.951-0.979) | 0.000 |
| unknown | 1.043 | (0.992-1.096) | 0.098 |  | 0.952 | (0.932-0.973) | 0.000 |
| SAPS 3 [per 10 points] x Weekday of admission | | | | | | | |
| Monday | 1.000 | (0.968-1.034) | 0.980 |  | 1.012 | (0.998-1.026) | 0.100 |
| Tuesday | 0.982 | (0.951-1.015) | 0.284 |  | 1.009 | (0.995-1.022) | 0.220 |
| *Wednesday* |  |  |  |  |  |  |  |
| Thursday | 0.995 | (0.961-1.029) | 0.756 |  | 1.009 | (0.996-1.023) | 0.181 |
| Friday | 0.981 | (0.948-1.015) | 0.264 |  | 0.999 | (0.986-1.013) | 0.939 |
| Saturday | 0.975 | (0.941-1.010) | 0.161 |  | 0.989 | (0.974-1.005) | 0.187 |
| Sunday | 0.973 | (0.940-1.008) | 0.132 |  | 0.982 | (0.966-0.998) | 0.026 |

# S5 Model for Hospital Mortality (n=147,397)

|  | Death in ICU (C=0.864) | | |  | ICU Discharge (C=0.718) | | |
| --- | --- | --- | --- | --- | --- | --- | --- |
|  | HR | 95% CI | p |  | HR | 95% CI | p |
| SAPS 3 [per 10 points] | 1.913 | (1.894-1.933) | 0.000 |  | 0.675 | (0.672-0.678) | 0.000 |
| Year of admission |  |  |  |  |  |  |  |
| *2012* | 1.000 |  |  |  | 1.000 |  |  |
| 2013 | 1.001 | (0.954-1.051) | 0.956 |  | 1.022 | (1.007-1.038) | 0.004 |
| 2014 | 0.961 | (0.912-1.014) | 0.144 |  | 1.076 | (1.058-1.095) | 0.000 |
| 2015 | 0.913 | (0.866-0.962) | 0.001 |  | 1.119 | (1.100-1.139) | 0.000 |
| Month of admission |  |  |  |  |  |  |  |
| *January* | 1.000 |  |  |  | 1.000 |  |  |
| February | 1.045 | (0.964-1.133) | 0.281 |  | 0.965 | (0.940-0.991) | 0.008 |
| March | 1.003 | (0.925-1.086) | 0.950 |  | 0.991 | (0.966-1.018) | 0.515 |
| April | 0.956 | (0.880-1.038) | 0.285 |  | 1.003 | (0.977-1.029) | 0.837 |
| May | 0.998 | (0.918-1.085) | 0.962 |  | 1.007 | (0.981-1.034) | 0.587 |
| June | 1.011 | (0.931-1.099) | 0.789 |  | 1.043 | (1.016-1.070) | 0.002 |
| July | 0.960 | (0.883-1.043) | 0.333 |  | 1.038 | (1.011-1.065) | 0.005 |
| August | 1.072 | (0.988-1.164) | 0.095 |  | 0.983 | (0.957-1.009) | 0.200 |
| September | 1.007 | (0.927-1.094) | 0.866 |  | 1.001 | (0.975-1.027) | 0.969 |
| October | 0.950 | (0.875-1.032) | 0.223 |  | 1.000 | (0.975-1.026) | 0.990 |
| November | 0.983 | (0.904-1.069) | 0.688 |  | 1.022 | (0.995-1.049) | 0.107 |
| December | 1.022 | (0.941-1.109) | 0.611 |  | 1.049 | (1.021-1.078) | 0.000 |
| Admission Type |  |  |  |  |  |  |  |
| *medical* | 1.000 |  |  |  | 1.000 |  |  |
| unscheduled surgery | 0.755 | (0.718-0.795) | 0.000 |  | 0.867 | (0.850-0.884) | 0.000 |
| scheduled surgery | 0.422 | (0.389-0.458) | 0.000 |  | 0.932 | (0.915-0.949) | 0.000 |
| surgery unspecified | 1.344 | (1.207-1.497) | 0.000 |  | 0.853 | (0.826-0.881) | 0.000 |
| unknown | 1.382 | (1.229-1.555) | 0.000 |  | 0.771 | (0.747-0.795) | 0.000 |
| Weekday of admission |  |  |  |  |  |  |  |
| Monday | 1.055 | (0.990-1.124) | 0.101 |  | 0.964 | (0.945-0.982) | 0.000 |
| Tuesday | 0.967 | (0.907-1.030) | 0.291 |  | 0.990 | (0.971-1.009) | 0.284 |
| *Wednesday* | 1.000 |  |  |  | 1.000 |  |  |
| Thursday | 0.946 | (0.888-1.009) | 0.091 |  | 0.975 | (0.956-0.993) | 0.008 |
| Friday | 1.045 | (0.980-1.114) | 0.175 |  | 1.064 | (1.044-1.085) | 0.000 |
| Saturday | 1.151 | (1.076-1.232) | 0.000 |  | 1.164 | (1.137-1.192) | 0.000 |
| Sunday | 1.105 | (1.032-1.183) | 0.004 |  | 0.961 | (0.938-0.985) | 0.001 |
| Weekday of event |  |  |  |  |  |  |  |
| Monday | 0.989 | (0.926-1.055) | 0.733 |  | 1.031 | (1.011-1.051) | 0.002 |
| Tuesday | 1.029 | (0.966-1.096) | 0.383 |  | 0.999 | (0.980-1.018) | 0.931 |
| *Wednesday* |  |  |  |  | 1.000 |  |  |
| Thursday | 1.027 | (0.964-1.095) | 0.405 |  | 0.919 | (0.901-0.937) | 0.000 |
| Friday | 1.009 | (0.946-1.076) | 0.780 |  | 1.151 | (1.130-1.173) | 0.000 |
| Saturday | 0.935 | (0.875-0.999) | 0.045 |  | 0.571 | (0.558-0.584) | 0.000 |
| Sunday | 0.854 | (0.798-0.914) | 0.000 |  | 0.335 | (0.326-0.344) | 0.000 |

# S6 Model for Patients Admitted to ICUs with at least 99% Documented Hospital Outcome (n=113,161)

|  | Death in ICU (C=0.862) | | |  | ICU Discharge (C=0.728) | | |
| --- | --- | --- | --- | --- | --- | --- | --- |
|  | HR | 95% CI | p |  | HR | 95% CI | p |
| SAPS 3 [per 10 points] | 1.896 | (1.874-1.917) | 0.000 |  | 0.680 | (0.676-0.683) | 0.000 |
| Year of admission |  |  |  |  |  |  |  |
| *2012* | 1.000 |  |  |  | 1.000 |  |  |
| 2013 | 0.991 | (0.940-1.045) | 0.746 |  | 1.003 | (0.986-1.020) | 0.744 |
| 2014 | 0.972 | (0.916-1.032) | 0.351 |  | 1.068 | (1.047-1.090) | 0.000 |
| 2015 | 0.924 | (0.871-0.980) | 0.009 |  | 1.103 | (1.081-1.126) | 0.000 |
| Month of admission |  |  |  |  |  |  |  |
| *January* | 1.000 |  |  |  | 1.000 |  |  |
| February | 1.024 | (0.937-1.120) | 0.600 |  | 0.974 | (0.945-1.004) | 0.088 |
| March | 0.946 | (0.865-1.035) | 0.226 |  | 1.003 | (0.973-1.033) | 0.861 |
| April | 0.944 | (0.862-1.035) | 0.222 |  | 0.998 | (0.968-1.028) | 0.875 |
| May | 0.970 | (0.884-1.064) | 0.518 |  | 1.006 | (0.977-1.037) | 0.674 |
| June | 0.994 | (0.907-1.089) | 0.897 |  | 1.019 | (0.989-1.050) | 0.218 |
| July | 0.959 | (0.874-1.051) | 0.368 |  | 1.014 | (0.985-1.045) | 0.347 |
| August | 1.051 | (0.959-1.152) | 0.284 |  | 0.981 | (0.951-1.011) | 0.213 |
| September | 1.000 | (0.912-1.096) | 0.999 |  | 0.995 | (0.966-1.026) | 0.752 |
| October | 0.905 | (0.825-0.993) | 0.035 |  | 1.001 | (0.972-1.031) | 0.960 |
| November | 0.957 | (0.872-1.050) | 0.353 |  | 0.994 | (0.965-1.024) | 0.703 |
| December | 0.953 | (0.869-1.045) | 0.305 |  | 0.985 | (0.956-1.016) | 0.339 |
| Admission Type |  |  |  |  |  |  |  |
| *medical* | 1.000 |  |  |  | 1.000 |  |  |
| unscheduled surgery | 0.768 | (0.725-0.814) | 0.000 |  | 0.926 | (0.905-0.947) | 0.000 |
| scheduled surgery | 0.423 | (0.385-0.464) | 0.000 |  | 1.183 | (1.158-1.208) | 0.000 |
| surgery unspecified | 1.322 | (1.184-1.475) | 0.000 |  | 0.915 | (0.885-0.946) | 0.000 |
| unknown | 1.494 | (1.290-1.730) | 0.000 |  | 1.011 | (0.968-1.056) | 0.610 |
| Weekday of admission |  |  |  |  |  |  |  |
| Monday | 1.068 | (0.995-1.148) | 0.069 |  | 0.927 | (0.906-0.949) | 0.000 |
| Tuesday | 0.962 | (0.896-1.033) | 0.285 |  | 0.982 | (0.960-1.004) | 0.107 |
| *Wednesday* | 1.000 |  |  |  | 1.000 |  |  |
| Thursday | 0.932 | (0.867-1.001) | 0.053 |  | 1.019 | (0.997-1.042) | 0.095 |
| Friday | 1.034 | (0.962-1.110) | 0.366 |  | 1.082 | (1.057-1.108) | 0.000 |
| Saturday | 1.167 | (1.082-1.259) | 0.000 |  | 0.962 | (0.935-0.990) | 0.008 |
| Sunday | 1.101 | (1.020-1.189) | 0.014 |  | 0.879 | (0.854-0.905) | 0.000 |
| Weekday of event |  |  |  |  |  |  |  |
| Monday | 1.003 | (0.933-1.079) | 0.926 |  | 1.017 | (0.992-1.042) | 0.175 |
| Tuesday | 1.040 | (0.969-1.116) | 0.279 |  | 1.029 | (1.006-1.052) | 0.015 |
| *Wednesday* | 1.000 |  |  |  | 1.000 |  |  |
| Thursday | 1.023 | (0.952-1.098) | 0.537 |  | 0.973 | (0.952-0.995) | 0.018 |
| Friday | 1.003 | (0.933-1.078) | 0.931 |  | 0.980 | (0.958-1.003) | 0.095 |
| Saturday | 0.940 | (0.873-1.013) | 0.104 |  | 0.594 | (0.579-0.610) | 0.000 |
| Sunday | 0.845 | (0.783-0.912) | 0.000 |  | 0.527 | (0.512-0.542) | 0.000 |

# S7 Models for SAPS 3 Tertiles

|  | **1^st^ Tertile (n=48,645)** | | | | | | |
| --- | --- | --- | --- | --- | --- | --- | --- |
|  | Death in ICU (C=0.827) | | |  | ICU Discharge (C=0.691) | | |
|  | HR | 95% CI | p |  | HR | 95% CI | p |
| SAPS 3 [per 10 points] | 1.938 | (1.599-2.348) | 0.000 |  | 0.816 | (0.801-0.830) | 0.000 |
| Year of admission |  |  |  |  |  |  |  |
| *2012* | 1.000 |  |  |  | 1.000 |  |  |
| 2013 | 1.072 | (0.861-1.335) | 0.536 |  | 0.997 | (0.973-1.022) | 0.813 |
| 2014 | 0.662 | (0.491-0.892) | 0.007 |  | 1.085 | (1.054-1.117) | 0.000 |
| 2015 | 0.572 | (0.404-0.809) | 0.002 |  | 1.114 | (1.081-1.148) | 0.000 |
| Month of admission |  |  |  |  |  |  |  |
| *January* | 1.000 |  |  |  | 1.000 |  |  |
| February | 1.332 | (0.911-1.947) | 0.139 |  | 0.974 | (0.932-1.017) | 0.234 |
| March | 1.060 | (0.708-1.586) | 0.778 |  | 0.986 | (0.944-1.030) | 0.536 |
| April | 1.048 | (0.698-1.574) | 0.820 |  | 0.993 | (0.951-1.038) | 0.764 |
| May | 1.042 | (0.685-1.583) | 0.848 |  | 0.981 | (0.939-1.025) | 0.388 |
| June | 0.601 | (0.376-0.961) | 0.034 |  | 1.010 | (0.967-1.055) | 0.643 |
| July | 0.588 | (0.365-0.946) | 0.029 |  | 1.025 | (0.981-1.070) | 0.271 |
| August | 0.961 | (0.630-1.465) | 0.852 |  | 0.995 | (0.951-1.040) | 0.819 |
| September | 0.813 | (0.525-1.258) | 0.352 |  | 1.018 | (0.974-1.063) | 0.431 |
| October | 0.870 | (0.566-1.337) | 0.525 |  | 1.011 | (0.968-1.056) | 0.620 |
| November | 0.755 | (0.486-1.173) | 0.211 |  | 1.021 | (0.977-1.066) | 0.357 |
| December | 0.745 | (0.472-1.176) | 0.206 |  | 0.991 | (0.947-1.036) | 0.689 |
| Admission Type |  |  |  |  |  |  |  |
| *medical* | 1.000 |  |  |  | 1.000 |  |  |
| unscheduled surgery | 0.644 | (0.441-0.939) | 0.022 |  | 0.847 | (0.811-0.884) | 0.000 |
| scheduled surgery | 0.346 | (0.251-0.476) | 0.000 |  | 1.156 | (1.118-1.195) | 0.000 |
| surgery unspecified | 1.112 | (0.511-2.420) | 0.789 |  | 1.061 | (1.000-1.126) | 0.049 |
| unknown | 1.299 | (0.964-1.750) | 0.085 |  | 1.374 | (1.319-1.432) | 0.000 |
| Weekday of admission |  |  |  |  |  |  |  |
| Monday | 1.340 | (0.959-1.873) | 0.087 |  | 0.952 | (0.918-0.988) | 0.009 |
| Tuesday | 1.123 | (0.796-1.585) | 0.509 |  | 0.982 | (0.950-1.015) | 0.290 |
| *Wednesday* | 1.000 |  |  |  | 1.000 |  |  |
| Thursday | 1.059 | (0.752-1.490) | 0.744 |  | 0.982 | (0.950-1.015) | 0.283 |
| Friday | 1.328 | (0.951-1.853) | 0.096 |  | 1.040 | (1.002-1.078) | 0.037 |
| Saturday | 1.770 | (1.235-2.537) | 0.002 |  | 0.967 | (0.923-1.013) | 0.158 |
| Sunday | 1.538 | (1.052-2.247) | 0.026 |  | 0.955 | (0.910-1.001) | 0.056 |
| Weekday of event |  |  |  |  |  |  |  |
| Monday | 0.950 | (0.676-1.336) | 0.770 |  | 0.972 | (0.934-1.011) | 0.158 |
| Tuesday | 0.789 | (0.553-1.125) | 0.191 |  | 1.023 | (0.989-1.059) | 0.188 |
| *Wednesday* | 1.000 |  |  |  | 1.000 |  |  |
| Thursday | 0.904 | (0.641-1.274) | 0.563 |  | 0.987 | (0.954-1.021) | 0.445 |
| Friday | 0.925 | (0.659-1.299) | 0.652 |  | 1.018 | (0.982-1.055) | 0.325 |
| Saturday | 1.044 | (0.748-1.459) | 0.799 |  | 0.708 | (0.680-0.737) | 0.000 |
| Sunday | 1.114 | (0.797-1.556) | 0.528 |  | 0.632 | (0.604-0.661) | 0.000 |

|  | **2^nd^ Tertile (n=50,842)** | | | | | | |
| --- | --- | --- | --- | --- | --- | --- | --- |
|  | Death in ICU (C=0.752) | | |  | ICU Discharge (C=0.644) | | |
|  | HR | 95% CI | p |  | HR | 95% CI | p |
| SAPS 3 [per 10 points] | 2.566 | (2.241-2.938) | 0.000 |  | 0.699 | (0.683-0.715) | 0.000 |
| Year of admission |  |  |  |  |  |  |  |
| *2012* | 1.000 |  |  |  | 1.000 |  |  |
| 2013 | 1.123 | (0.980-1.287) | 0.095 |  | 0.974 | (0.951-0.999) | 0.038 |
| 2014 | 0.893 | (0.757-1.054) | 0.182 |  | 1.009 | (0.981-1.038) | 0.524 |
| 2015 | 0.856 | (0.720-1.017) | 0.076 |  | 1.047 | (1.017-1.077) | 0.002 |
| Month of admission |  |  |  |  |  |  |  |
| *January* | 1.000 |  |  |  | 1.000 |  |  |
| February | 1.346 | (1.039-1.744) | 0.024 |  | 1.006 | (0.963-1.051) | 0.790 |
| March | 1.023 | (0.781-1.340) | 0.869 |  | 1.026 | (0.983-1.071) | 0.243 |
| April | 1.164 | (0.892-1.519) | 0.263 |  | 1.003 | (0.960-1.048) | 0.887 |
| May | 1.053 | (0.800-1.386) | 0.713 |  | 1.020 | (0.977-1.065) | 0.375 |
| June | 1.246 | (0.960-1.618) | 0.099 |  | 1.017 | (0.974-1.062) | 0.441 |
| July | 1.157 | (0.887-1.510) | 0.282 |  | 0.984 | (0.943-1.027) | 0.470 |
| August | 1.213 | (0.935-1.574) | 0.147 |  | 0.994 | (0.952-1.038) | 0.794 |
| September | 1.086 | (0.831-1.418) | 0.545 |  | 1.005 | (0.963-1.050) | 0.810 |
| October | 1.273 | (0.985-1.644) | 0.065 |  | 0.992 | (0.951-1.034) | 0.696 |
| November | 1.262 | (0.974-1.635) | 0.079 |  | 0.987 | (0.946-1.031) | 0.562 |
| December | 1.265 | (0.974-1.642) | 0.078 |  | 0.972 | (0.930-1.015) | 0.203 |
| Admission Type |  |  |  |  |  |  |  |
| *medical* | 1.000 |  |  |  | 1.000 |  |  |
| unscheduled surgery | 0.622 | (0.516-0.750) | 0.000 |  | 0.900 | (0.871-0.930) | 0.000 |
| scheduled surgery | 0.392 | (0.327-0.470) | 0.000 |  | 1.241 | (1.206-1.277) | 0.000 |
| surgery unspecified | 1.763 | (1.298-2.395) | 0.000 |  | 0.853 | (0.812-0.897) | 0.000 |
| unknown | 1.053 | (0.763-1.453) | 0.753 |  | 0.903 | (0.840-0.971) | 0.006 |
| Weekday of admission |  |  |  |  |  |  |  |
| Monday | 1.042 | (0.862-1.261) | 0.669 |  | 0.938 | (0.907-0.971) | 0.000 |
| Tuesday | 0.959 | (0.794-1.160) | 0.668 |  | 0.988 | (0.957-1.020) | 0.462 |
| *Wednesday* | 1.000 |  |  |  | 1.000 |  |  |
| Thursday | 0.948 | (0.782-1.148) | 0.583 |  | 1.023 | (0.991-1.057) | 0.157 |
| Friday | 1.191 | (0.990-1.434) | 0.064 |  | 1.042 | (1.007-1.078) | 0.017 |
| Saturday | 1.394 | (1.138-1.707) | 0.001 |  | 0.990 | (0.950-1.031) | 0.614 |
| Sunday | 1.029 | (0.826-1.282) | 0.799 |  | 0.916 | (0.879-0.955) | 0.000 |
| Weekday of event |  |  |  |  |  |  |  |
| Monday | 1.109 | (0.912-1.347) | 0.300 |  | 1.037 | (1.001-1.074) | 0.045 |
| Tuesday | 1.063 | (0.875-1.290) | 0.539 |  | 1.022 | (0.989-1.056) | 0.193 |
| *Wednesday* | 1.000 |  |  |  | 1.000 |  |  |
| Thursday | 0.985 | (0.809-1.199) | 0.883 |  | 0.953 | (0.923-0.985) | 0.004 |
| Friday | 0.986 | (0.810-1.201) | 0.892 |  | 0.988 | (0.955-1.022) | 0.483 |
| Saturday | 0.926 | (0.757-1.131) | 0.450 |  | 0.623 | (0.600-0.647) | 0.000 |
| Sunday | 0.992 | (0.811-1.213) | 0.936 |  | 0.561 | (0.538-0.584) | 0.000 |

|  | **3^rd^ Tertile (n=51,781)** | | | | | | |
| --- | --- | --- | --- | --- | --- | --- | --- |
|  | Death in ICU (C=0.748) | | |  | ICU Discharge (C=0.688) | | |
|  | HR | 95% CI | p |  | HR | 95% CI | p |
| SAPS 3 [per 10 points] | 1.741 | (1.718-1.764) | 0.000 |  | 0.633 | (0.626-0.640) | 0.000 |
| Year of admission |  |  |  |  |  |  |  |
| *2012* | 1.000 |  |  |  | 1.000 |  |  |
| 2013 | 1.004 | (0.952-1.059) | 0.880 |  | 0.986 | (0.958-1.015) | 0.352 |
| 2014 | 1.023 | (0.966-1.084) | 0.434 |  | 0.988 | (0.957-1.019) | 0.439 |
| 2015 | 0.957 | (0.904-1.013) | 0.128 |  | 1.036 | (1.005-1.069) | 0.025 |
| Month of admission |  |  |  |  |  |  |  |
| *January* | 1.000 |  |  |  | 1.000 |  |  |
| February | 1.004 | (0.920-1.095) | 0.936 |  | 0.972 | (0.925-1.020) | 0.248 |
| March | 1.002 | (0.920-1.092) | 0.956 |  | 0.994 | (0.948-1.043) | 0.818 |
| April | 0.941 | (0.861-1.029) | 0.181 |  | 1.010 | (0.962-1.060) | 0.696 |
| May | 1.003 | (0.918-1.097) | 0.940 |  | 1.012 | (0.964-1.062) | 0.633 |
| June | 1.023 | (0.936-1.118) | 0.617 |  | 0.985 | (0.938-1.035) | 0.551 |
| July | 0.963 | (0.881-1.053) | 0.412 |  | 1.017 | (0.969-1.067) | 0.487 |
| August | 1.071 | (0.980-1.170) | 0.131 |  | 0.954 | (0.909-1.003) | 0.063 |
| September | 1.031 | (0.944-1.127) | 0.493 |  | 0.978 | (0.931-1.027) | 0.364 |
| October | 0.941 | (0.860-1.029) | 0.182 |  | 0.988 | (0.942-1.037) | 0.636 |
| November | 0.981 | (0.896-1.073) | 0.673 |  | 0.957 | (0.911-1.005) | 0.078 |
| December | 0.981 | (0.898-1.072) | 0.677 |  | 0.991 | (0.944-1.040) | 0.718 |
| Admission Type |  |  |  |  |  |  |  |
| *medical* | 1.000 |  |  |  | 1.000 |  |  |
| unscheduled surgery | 0.735 | (0.697-0.776) | 0.000 |  | 1.022 | (0.992-1.052) | 0.147 |
| scheduled surgery | 0.557 | (0.504-0.615) | 0.000 |  | 1.359 | (1.310-1.410) | 0.000 |
| surgery unspecified | 1.435 | (1.276-1.613) | 0.000 |  | 0.763 | (0.715-0.814) | 0.000 |
| unknown | 1.400 | (1.194-1.640) | 0.000 |  | 0.868 | (0.790-0.953) | 0.003 |
| Weekday of admission |  |  |  |  |  |  |  |
| Monday | 1.042 | (0.972-1.117) | 0.243 |  | 0.945 | (0.910-0.980) | 0.003 |
| Tuesday | 0.974 | (0.910-1.043) | 0.452 |  | 0.992 | (0.957-1.029) | 0.663 |
| *Wednesday* | 1.000 |  |  |  | 1.000 |  |  |
| Thursday | 0.931 | (0.869-0.998) | 0.045 |  | 1.048 | (1.011-1.087) | 0.011 |
| Friday | 1.004 | (0.936-1.077) | 0.908 |  | 1.078 | (1.039-1.118) | 0.000 |
| Saturday | 1.093 | (1.015-1.176) | 0.018 |  | 0.999 | (0.959-1.040) | 0.946 |
| Sunday | 1.089 | (1.012-1.171) | 0.023 |  | 0.915 | (0.877-0.953) | 0.000 |
| Weekday of event |  |  |  |  |  |  |  |
| Monday | 0.976 | (0.909-1.047) | 0.496 |  | 1.019 | (0.982-1.056) | 0.318 |
| Tuesday | 1.033 | (0.965-1.105) | 0.356 |  | 1.007 | (0.971-1.043) | 0.719 |
| *Wednesday* | 1.000 |  |  |  | 1.000 |  |  |
| Thursday | 1.037 | (0.969-1.111) | 0.293 |  | 0.981 | (0.946-1.016) | 0.281 |
| Friday | 1.017 | (0.948-1.090) | 0.642 |  | 0.998 | (0.963-1.034) | 0.905 |
| Saturday | 0.934 | (0.869-1.003) | 0.062 |  | 0.559 | (0.537-0.583) | 0.000 |
| Sunday | 0.829 | (0.770-0.893) | 0.000 |  | 0.500 | (0.479-0.523) | 0.000 |

# S8 Model for Readmissions Only (n=14,252)

|  | Death in ICU (C=0.788) | | |  | ICU Discharge (C=0.710) | | |
| --- | --- | --- | --- | --- | --- | --- | --- |
|  | HR | 95% CI | p |  | HR | 95% CI | p |
| SAPS 3 [per 10 points] | 1.724 | (1.671-1.780) | 0.000 |  | 0.727 | (0.717-0.738) | 0.000 |
| Year of admission |  |  |  |  |  |  |  |
| *2012* | 1.000 |  |  |  | 1.000 |  |  |
| 2013 | 1.065 | (0.920-1.232) | 0.400 |  | 1.027 | (0.976-1.081) | 0.300 |
| 2014 | 0.896 | (0.758-1.058) | 0.195 |  | 1.058 | (0.998-1.122) | 0.060 |
| 2015 | 0.927 | (0.788-1.090) | 0.358 |  | 1.082 | (1.020-1.147) | 0.009 |
| Month of admission |  |  |  |  |  |  |  |
| *January* | 1.000 |  |  |  | 1.000 |  |  |
| February | 0.863 | (0.670-1.113) | 0.256 |  | 1.020 | (0.930-1.119) | 0.674 |
| March | 0.975 | (0.763-1.246) | 0.841 |  | 1.026 | (0.937-1.123) | 0.586 |
| April | 0.838 | (0.646-1.087) | 0.183 |  | 1.079 | (0.984-1.183) | 0.104 |
| May | 0.901 | (0.698-1.163) | 0.425 |  | 0.994 | (0.906-1.090) | 0.893 |
| June | 1.020 | (0.793-1.311) | 0.878 |  | 1.016 | (0.926-1.115) | 0.740 |
| July | 0.904 | (0.707-1.157) | 0.423 |  | 1.065 | (0.974-1.165) | 0.166 |
| August | 0.910 | (0.709-1.168) | 0.459 |  | 0.978 | (0.892-1.071) | 0.626 |
| September | 0.894 | (0.690-1.158) | 0.395 |  | 1.056 | (0.964-1.156) | 0.242 |
| October | 0.980 | (0.763-1.260) | 0.877 |  | 1.066 | (0.973-1.168) | 0.168 |
| November | 0.845 | (0.654-1.093) | 0.199 |  | 1.064 | (0.971-1.167) | 0.184 |
| December | 1.097 | (0.852-1.413) | 0.472 |  | 1.076 | (0.980-1.181) | 0.126 |
| Admission Type |  |  |  |  |  |  |  |
| *medical* | 1.000 |  |  |  | 1.000 |  |  |
| unscheduled surgery | 0.821 | (0.709-0.949) | 0.008 |  | 0.964 | (0.911-1.019) | 0.195 |
| scheduled surgery | 0.803 | (0.663-0.972) | 0.025 |  | 1.121 | (1.060-1.184) | 0.000 |
| surgery unspecified | 1.421 | (1.085-1.861) | 0.011 |  | 0.851 | (0.773-0.937) | 0.001 |
| unknown | 1.234 | (0.889-1.714) | 0.208 |  | 1.171 | (1.076-1.274) | 0.000 |
| Weekday of admission |  |  |  |  |  |  |  |
| Monday | 1.048 | (0.866-1.269) | 0.628 |  | 0.900 | (0.843-0.961) | 0.002 |
| Tuesday | 1.156 | (0.960-1.391) | 0.125 |  | 0.944 | (0.884-1.007) | 0.083 |
| *Wednesday* | 1.000 |  |  |  | 1.000 |  |  |
| Thursday | 1.093 | (0.907-1.318) | 0.350 |  | 0.960 | (0.900-1.024) | 0.211 |
| Friday | 1.106 | (0.919-1.332) | 0.287 |  | 1.052 | (0.987-1.123) | 0.122 |
| Saturday | 0.997 | (0.809-1.227) | 0.974 |  | 1.028 | (0.956-1.106) | 0.451 |
| Sunday | 1.350 | (1.106-1.649) | 0.003 |  | 0.876 | (0.811-0.946) | 0.001 |
| Weekday of event |  |  |  |  |  |  |  |
| Monday | 1.064 | (0.883-1.282) | 0.514 |  | 1.073 | (1.008-1.142) | 0.028 |
| Tuesday | 0.990 | (0.822-1.193) | 0.918 |  | 0.974 | (0.915-1.037) | 0.414 |
| *Wednesday* | 1.000 |  |  |  | 1.000 |  |  |
| Thursday | 0.970 | (0.805-1.168) | 0.745 |  | 0.908 | (0.852-0.968) | 0.003 |
| Friday | 0.997 | (0.828-1.201) | 0.979 |  | 0.942 | (0.884-1.004) | 0.066 |
| Saturday | 0.922 | (0.761-1.117) | 0.405 |  | 0.469 | (0.435-0.507) | 0.000 |
| Sunday | 0.828 | (0.678-1.010) | 0.062 |  | 0.459 | (0.425-0.497) | 0.000 |

# S9 Models For Different Admission Types

|  | **Medical (n=55,356)** | | | | | | |
| --- | --- | --- | --- | --- | --- | --- | --- |
|  | Death in ICU (C=0.820) | | |  | ICU Discharge (C=0.737) | | |
|  | HR | 95% CI | p |  | HR | 95% CI | p |
| SAPS 3 [per 10 points] | 1.823 | (1.801-1.846) | 0.000 |  | 0.649 | (0.645-0.654) | 0.000 |
| Year of admission |  |  |  |  |  |  |  |
| *2012* | 1.000 |  |  |  | 1.000 |  |  |
| 2013 | 1.035 | (0.970-1.104) | 0.302 |  | 0.972 | (0.946-0.998) | 0.035 |
| 2014 | 0.983 | (0.921-1.050) | 0.614 |  | 1.044 | (1.015-1.073) | 0.003 |
| 2015 | 0.910 | (0.852-0.971) | 0.005 |  | 1.142 | (1.110-1.175) | 0.000 |
| Month of admission |  |  |  |  |  |  |  |
| *January* | 1.000 |  |  |  | 1.000 |  |  |
| February | 1.030 | (0.931-1.140) | 0.564 |  | 0.970 | (0.927-1.014) | 0.179 |
| March | 1.003 | (0.907-1.109) | 0.954 |  | 0.974 | (0.932-1.018) | 0.243 |
| April | 0.986 | (0.890-1.093) | 0.791 |  | 0.990 | (0.947-1.034) | 0.647 |
| May | 0.984 | (0.885-1.093) | 0.763 |  | 1.042 | (0.997-1.090) | 0.069 |
| June | 1.038 | (0.936-1.152) | 0.480 |  | 1.002 | (0.959-1.048) | 0.914 |
| July | 0.954 | (0.859-1.059) | 0.377 |  | 1.019 | (0.975-1.064) | 0.409 |
| August | 1.045 | (0.942-1.161) | 0.405 |  | 1.013 | (0.968-1.059) | 0.584 |
| September | 1.031 | (0.929-1.144) | 0.564 |  | 0.984 | (0.941-1.029) | 0.475 |
| October | 0.936 | (0.842-1.039) | 0.215 |  | 1.010 | (0.966-1.055) | 0.666 |
| November | 0.998 | (0.898-1.108) | 0.964 |  | 0.976 | (0.934-1.021) | 0.293 |
| December | 0.997 | (0.899-1.105) | 0.954 |  | 0.969 | (0.927-1.013) | 0.161 |
| Weekday of admission |  |  |  |  |  |  |  |
| Monday | 1.044 | (0.963-1.133) | 0.298 |  | 0.930 | (0.898-0.962) | 0.000 |
| Tuesday | 0.993 | (0.916-1.076) | 0.859 |  | 0.975 | (0.943-1.008) | 0.137 |
| *Wednesday* | 1.000 |  |  |  | 1.000 |  |  |
| Thursday | 0.938 | (0.865-1.018) | 0.127 |  | 1.025 | (0.992-1.061) | 0.143 |
| Friday | 1.050 | (0.967-1.141) | 0.244 |  | 1.050 | (1.014-1.087) | 0.007 |
| Saturday | 1.129 | (1.037-1.229) | 0.005 |  | 1.062 | (1.024-1.103) | 0.001 |
| Sunday | 1.096 | (1.007-1.194) | 0.034 |  | 1.009 | (0.971-1.047) | 0.656 |
| Weekday of event |  |  |  |  |  |  |  |
| Monday | 0.994 | (0.916-1.079) | 0.894 |  | 0.924 | (0.894-0.956) | 0.000 |
| Tuesday | 0.998 | (0.921-1.081) | 0.954 |  | 0.969 | (0.938-1.001) | 0.058 |
| *Wednesday* | 1.000 |  |  |  | 1.000 |  |  |
| Thursday | 1.050 | (0.970-1.137) | 0.227 |  | 0.951 | (0.920-0.982) | 0.002 |
| Friday | 0.953 | (0.877-1.035) | 0.252 |  | 0.977 | (0.945-1.010) | 0.168 |
| Saturday | 0.931 | (0.856-1.013) | 0.096 |  | 0.597 | (0.574-0.620) | 0.000 |
| Sunday | 0.872 | (0.800-0.950) | 0.002 |  | 0.542 | (0.521-0.564) | 0.000 |

|  | **Unscheduled Surgery (n=23,180)** | | | | | | |
| --- | --- | --- | --- | --- | --- | --- | --- |
|  | Death in ICU (C=0.816) | | |  | ICU Discharge (C=0.703) | | |
|  | HR | 95% CI | p |  | HR | 95% CI | p |
| SAPS 3 [per 10 points] | 1.981 | (1.930-2.033) | 0.000 |  | 0.696 | (0.689-0.704) | 0.000 |
| Year of admission |  |  |  |  |  |  |  |
| *2012* | 1.000 |  |  |  | 1.000 |  |  |
| 2013 | 1.047 | (0.929-1.181) | 0.451 |  | 0.968 | (0.930-1.008) | 0.118 |
| 2014 | 1.062 | (0.938-1.203) | 0.341 |  | 1.035 | (0.992-1.080) | 0.116 |
| 2015 | 1.039 | (0.917-1.177) | 0.546 |  | 1.055 | (1.009-1.102) | 0.017 |
| Month of admission |  |  |  |  |  |  |  |
| *January* | 1.000 |  |  |  | 1.000 |  |  |
| February | 1.011 | (0.826-1.237) | 0.917 |  | 0.930 | (0.867-0.997) | 0.042 |
| March | 1.005 | (0.826-1.222) | 0.962 |  | 0.962 | (0.898-1.031) | 0.274 |
| April | 0.964 | (0.788-1.179) | 0.718 |  | 0.896 | (0.835-0.960) | 0.002 |
| May | 1.046 | (0.860-1.273) | 0.650 |  | 0.975 | (0.910-1.044) | 0.466 |
| June | 0.956 | (0.781-1.170) | 0.661 |  | 0.966 | (0.902-1.035) | 0.329 |
| July | 1.057 | (0.871-1.283) | 0.572 |  | 0.940 | (0.878-1.006) | 0.075 |
| August | 1.108 | (0.912-1.347) | 0.303 |  | 0.937 | (0.875-1.004) | 0.066 |
| September | 1.039 | (0.855-1.262) | 0.702 |  | 0.934 | (0.872-1.000) | 0.051 |
| October | 1.066 | (0.879-1.292) | 0.516 |  | 0.899 | (0.840-0.962) | 0.002 |
| November | 1.004 | (0.824-1.225) | 0.965 |  | 0.929 | (0.867-0.996) | 0.038 |
| December | 0.904 | (0.742-1.101) | 0.314 |  | 0.952 | (0.889-1.019) | 0.155 |
| Weekday of admission |  |  |  |  |  |  |  |
| Monday | 1.052 | (0.904-1.223) | 0.514 |  | 0.919 | (0.870-0.970) | 0.002 |
| Tuesday | 0.977 | (0.844-1.132) | 0.758 |  | 0.986 | (0.935-1.039) | 0.592 |
| *Wednesday* | 1.000 |  |  |  | 1.000 |  |  |
| Thursday | 0.931 | (0.801-1.082) | 0.350 |  | 1.055 | (1.002-1.111) | 0.043 |
| Friday | 1.054 | (0.912-1.218) | 0.480 |  | 1.061 | (1.007-1.119) | 0.025 |
| Saturday | 1.030 | (0.879-1.207) | 0.715 |  | 0.992 | (0.938-1.048) | 0.766 |
| Sunday | 1.022 | (0.870-1.200) | 0.793 |  | 0.920 | (0.869-0.975) | 0.005 |
| Weekday of event |  |  |  |  |  |  |  |
| Monday | 1.013 | (0.873-1.176) | 0.867 |  | 1.111 | (1.056-1.169) | 0.000 |
| Tuesday | 1.093 | (0.947-1.263) | 0.224 |  | 1.057 | (1.004-1.113) | 0.034 |
| *Wednesday* | 1.000 |  |  |  | 1.000 |  |  |
| Thursday | 0.916 | (0.789-1.064) | 0.249 |  | 1.023 | (0.971-1.077) | 0.392 |
| Friday | 1.091 | (0.944-1.260) | 0.240 |  | 1.013 | (0.962-1.068) | 0.619 |
| Saturday | 0.864 | (0.741-1.008) | 0.063 |  | 0.656 | (0.619-0.695) | 0.000 |
| Sunday | 0.743 | (0.632-0.873) | 0.000 |  | 0.581 | (0.547-0.616) | 0.000 |
|  |  |  |  |  |  |  |  |

|  | **Scheduled Surgery (n=49,237)** | | | | | | |
| --- | --- | --- | --- | --- | --- | --- | --- |
|  | Death in ICU (C=0.867) | | |  | ICU Discharge (C=0.723) | | |
|  | HR | 95% CI | p |  | HR | 95% CI | p |
| SAPS 3 [per 10 points] | 2.568 | (2.445-2.698) | 0.000 |  | 0.746 | (0.740-0.753) | 0.000 |
| Year of admission |  |  |  |  |  |  |  |
| *2012* | 1.000 |  |  |  | 1.000 |  |  |
| 2013 | 1.059 | (0.867-1.293) | 0.576 |  | 0.988 | (0.961-1.015) | 0.385 |
| 2014 | 1.017 | (0.832-1.243) | 0.872 |  | 0.982 | (0.955-1.009) | 0.192 |
| 2015 | 1.030 | (0.842-1.262) | 0.772 |  | 0.989 | (0.962-1.017) | 0.435 |
| Month of admission |  |  |  |  |  |  |  |
| *January* | 1.000 |  |  |  | 1.000 |  |  |
| February | 1.107 | (0.806-1.520) | 0.529 |  | 1.005 | (0.962-1.051) | 0.808 |
| March | 0.985 | (0.715-1.356) | 0.925 |  | 1.040 | (0.996-1.086) | 0.073 |
| April | 0.851 | (0.602-1.201) | 0.358 |  | 1.027 | (0.983-1.072) | 0.235 |
| May | 1.061 | (0.769-1.464) | 0.719 |  | 1.005 | (0.962-1.050) | 0.819 |
| June | 1.087 | (0.794-1.490) | 0.602 |  | 1.027 | (0.983-1.072) | 0.237 |
| July | 0.764 | (0.545-1.070) | 0.117 |  | 1.030 | (0.987-1.076) | 0.176 |
| August | 1.309 | (0.960-1.786) | 0.089 |  | 0.960 | (0.918-1.004) | 0.075 |
| September | 0.913 | (0.657-1.267) | 0.585 |  | 1.041 | (0.996-1.087) | 0.074 |
| October | 1.081 | (0.790-1.479) | 0.628 |  | 1.016 | (0.973-1.060) | 0.476 |
| November | 1.050 | (0.758-1.455) | 0.769 |  | 1.007 | (0.964-1.051) | 0.760 |
| December | 0.836 | (0.592-1.180) | 0.308 |  | 0.980 | (0.937-1.025) | 0.384 |
| Weekday of admission |  |  |  |  |  |  |  |
| Monday | 1.019 | (0.817-1.272) | 0.865 |  | 0.933 | (0.899-0.968) | 0.000 |
| Tuesday | 0.828 | (0.656-1.045) | 0.113 |  | 0.982 | (0.950-1.016) | 0.301 |
| *Wednesday* | 1.000 |  |  |  | 1.000 |  |  |
| Thursday | 1.047 | (0.840-1.305) | 0.682 |  | 0.980 | (0.948-1.014) | 0.244 |
| Friday | 1.020 | (0.813-1.281) | 0.863 |  | 1.094 | (1.055-1.134) | 0.000 |
| Saturday | 1.652 | (1.213-2.249) | 0.001 |  | 0.744 | (0.699-0.792) | 0.000 |
| Sunday | 1.466 | (1.047-2.051) | 0.026 |  | 0.706 | (0.661-0.754) | 0.000 |
| Weekday of event |  |  |  |  |  |  |  |
| Monday | 1.069 | (0.830-1.378) | 0.606 |  | 1.197 | (1.146-1.249) | 0.000 |
| Tuesday | 1.032 | (0.806-1.322) | 0.802 |  | 1.083 | (1.044-1.124) | 0.000 |
| *Wednesday* | 1.000 |  |  |  | 1.000 |  |  |
| Thursday | 1.023 | (0.801-1.307) | 0.855 |  | 0.966 | (0.933-1.001) | 0.057 |
| Friday | 1.168 | (0.920-1.484) | 0.203 |  | 0.993 | (0.957-1.030) | 0.707 |
| Saturday | 0.897 | (0.692-1.164) | 0.413 |  | 0.582 | (0.559-0.606) | 0.000 |
| Sunday | 0.938 | (0.719-1.223) | 0.635 |  | 0.497 | (0.474-0.522) | 0.000 |

|  | **Surgery Unspecified (n=16,287)** | | | | | | |
| --- | --- | --- | --- | --- | --- | --- | --- |
|  | Death in ICU (C=0.868) | | |  | ICU Discharge (C=0.697) | | |
|  | HR | 95% CI | p |  | HR | 95% CI | p |
| SAPS 3 [per 10 points] | 2.281 | (2.203-2.362) | 0.000 |  | 0.640 | (0.631-0.649) | 0.000 |
| Year of admission |  |  |  |  |  |  |  |
| *2012* | 1.000 |  |  |  | 1.000 |  |  |
| 2013 | 0.898 | (0.793-1.017) | 0.091 |  | 1.063 | (1.025-1.103) | 0.001 |
| 2014 | 0.745 | (0.124-4.480) | 0.748 |  | 1.541 | (0.735-3.229) | 0.252 |
| 2015 | 0.000 | (0.000-Inf) | 0.990 |  | 1.980 | (0.910-4.308) | 0.085 |
| Month of admission |  |  |  |  |  |  |  |
| *January* | 1.000 |  |  |  | 1.000 |  |  |
| February | 1.025 | (0.794-1.322) | 0.851 |  | 0.981 | (0.907-1.060) | 0.622 |
| March | 0.954 | (0.737-1.234) | 0.720 |  | 1.007 | (0.933-1.087) | 0.864 |
| April | 0.940 | (0.719-1.229) | 0.651 |  | 0.964 | (0.892-1.043) | 0.363 |
| May | 0.916 | (0.700-1.198) | 0.522 |  | 0.966 | (0.893-1.044) | 0.381 |
| June | 0.946 | (0.727-1.231) | 0.681 |  | 0.986 | (0.913-1.066) | 0.730 |
| July | 0.873 | (0.660-1.154) | 0.339 |  | 0.996 | (0.922-1.075) | 0.913 |
| August | 1.056 | (0.814-1.368) | 0.683 |  | 0.968 | (0.894-1.047) | 0.411 |
| September | 0.962 | (0.735-1.260) | 0.780 |  | 1.031 | (0.952-1.116) | 0.451 |
| October | 0.887 | (0.681-1.156) | 0.376 |  | 0.997 | (0.922-1.077) | 0.934 |
| November | 0.945 | (0.724-1.232) | 0.674 |  | 1.007 | (0.932-1.089) | 0.851 |
| December | 1.182 | (0.896-1.560) | 0.237 |  | 1.073 | (0.988-1.165) | 0.094 |
| Weekday of admission |  |  |  |  |  |  |  |
| Monday | 1.226 | (0.985-1.526) | 0.068 |  | 0.927 | (0.871-0.986) | 0.015 |
| Tuesday | 1.034 | (0.835-1.280) | 0.760 |  | 0.986 | (0.931-1.045) | 0.641 |
| *Wednesday* | 1.000 |  |  |  | 1.000 |  |  |
| Thursday | 0.922 | (0.740-1.148) | 0.468 |  | 1.049 | (0.990-1.112) | 0.107 |
| Friday | 0.977 | (0.786-1.215) | 0.836 |  | 1.053 | (0.991-1.119) | 0.096 |
| Saturday | 1.338 | (1.068-1.676) | 0.011 |  | 0.983 | (0.916-1.056) | 0.644 |
| Sunday | 1.123 | (0.892-1.415) | 0.322 |  | 0.943 | (0.878-1.013) | 0.107 |
| Weekday of event |  |  |  |  |  |  |  |
| Monday | 0.870 | (0.696-1.089) | 0.224 |  | 1.001 | (0.940-1.065) | 0.985 |
| Tuesday | 0.986 | (0.799-1.218) | 0.899 |  | 1.044 | (0.985-1.107) | 0.149 |
| *Wednesday* | 1.000 |  |  |  | 1.000 |  |  |
| Thursday | 1.095 | (0.888-1.350) | 0.396 |  | 0.970 | (0.915-1.028) | 0.300 |
| Friday | 1.024 | (0.826-1.269) | 0.832 |  | 1.003 | (0.944-1.066) | 0.922 |
| Saturday | 1.002 | (0.807-1.244) | 0.987 |  | 0.641 | (0.599-0.687) | 0.000 |
| Sunday | 0.840 | (0.669-1.055) | 0.134 |  | 0.590 | (0.549-0.634) | 0.000 |

|  | **Unknown (n=7,208)** | | | | | | |
| --- | --- | --- | --- | --- | --- | --- | --- |
|  | Death in ICU (C=0.863) | | |  | ICU Discharge (C=0.767) | | |
|  | HR | 95% CI | p |  | HR | 95% CI | p |
| SAPS 3 [per 10 points] | 1.924 | (1.799-2.057) | 0.000 |  | 0.683 | (0.666-0.700) | 0.000 |
| Year of admission |  |  |  |  |  |  |  |
| *2012* | 1.000 |  |  |  | 1.000 |  |  |
| 2013 | 1.055 | (0.809-1.376) | 0.692 |  | 1.040 | (0.978-1.106) | 0.211 |
| 2014 | 0.932 | (0.558-1.555) | 0.786 |  | 1.067 | (0.977-1.165) | 0.150 |
| 2015 | 1.167 | (0.687-1.981) | 0.568 |  | 1.191 | (1.087-1.306) | 0.000 |
| Month of admission |  |  |  |  |  |  |  |
| *January* | 1.000 |  |  |  | 1.000 |  |  |
| February | 2.161 | (1.340-3.486) | 0.002 |  | 0.955 | (0.849-1.075) | 0.446 |
| March | 2.002 | (1.224-3.276) | 0.006 |  | 0.922 | (0.818-1.038) | 0.179 |
| April | 1.673 | (0.982-2.850) | 0.058 |  | 1.070 | (0.949-1.207) | 0.270 |
| May | 1.982 | (1.123-3.498) | 0.018 |  | 0.908 | (0.802-1.027) | 0.125 |
| June | 1.418 | (0.830-2.421) | 0.202 |  | 1.016 | (0.904-1.141) | 0.793 |
| July | 1.836 | (1.099-3.068) | 0.020 |  | 1.014 | (0.902-1.140) | 0.813 |
| August | 1.706 | (1.007-2.891) | 0.047 |  | 0.970 | (0.862-1.091) | 0.609 |
| September | 1.145 | (0.637-2.058) | 0.651 |  | 1.045 | (0.928-1.176) | 0.472 |
| October | 1.175 | (0.659-2.096) | 0.584 |  | 1.044 | (0.926-1.178) | 0.479 |
| November | 1.200 | (0.671-2.145) | 0.539 |  | 1.016 | (0.902-1.144) | 0.794 |
| December | 1.745 | (1.051-2.897) | 0.031 |  | 1.022 | (0.910-1.148) | 0.713 |
| Weekday of admission |  |  |  |  |  |  |  |
| Monday | 1.355 | (0.911-2.014) | 0.133 |  | 1.040 | (0.943-1.146) | 0.431 |
| Tuesday | 0.873 | (0.575-1.325) | 0.524 |  | 0.983 | (0.898-1.075) | 0.703 |
| *Wednesday* | 1.000 |  |  |  | 1.000 |  |  |
| Thursday | 0.837 | (0.550-1.274) | 0.407 |  | 0.978 | (0.894-1.070) | 0.623 |
| Friday | 1.362 | (0.915-2.029) | 0.128 |  | 1.001 | (0.909-1.102) | 0.989 |
| Saturday | 1.247 | (0.805-1.930) | 0.323 |  | 1.111 | (0.996-1.239) | 0.059 |
| Sunday | 1.244 | (0.798-1.938) | 0.335 |  | 1.067 | (0.956-1.190) | 0.249 |
| Weekday of event |  |  |  |  |  |  |  |
| Monday | 0.858 | (0.557-1.322) | 0.487 |  | 0.890 | (0.806-0.982) | 0.021 |
| Tuesday | 1.338 | (0.907-1.974) | 0.143 |  | 0.986 | (0.901-1.078) | 0.750 |
| *Wednesday* | 1.000 |  |  |  | 1.000 |  |  |
| Thursday | 1.126 | (0.748-1.695) | 0.570 |  | 0.996 | (0.912-1.088) | 0.936 |
| Friday | 1.385 | (0.933-2.057) | 0.106 |  | 1.075 | (0.979-1.180) | 0.129 |
| Saturday | 1.286 | (0.858-1.927) | 0.223 |  | 0.678 | (0.609-0.754) | 0.000 |
| Sunday | 0.961 | (0.623-1.481) | 0.856 |  | 0.706 | (0.634-0.786) | 0.000 |

# S10 Main Analysis with Cox Proportional Hazards Model (n=151,268)

|  | Death in ICU (C=0.846) | | |  | ICU Discharge (C=0.723) | | |
| --- | --- | --- | --- | --- | --- | --- | --- |
|  | HR | 95% CI | p |  | HR | 95% CI | p |
| SAPS 3 [per 10 points] | 1.665 | (1.648-1.683) | 0.000 |  | 0.720 | (0.716-0.723) | 0.000 |
| Year of admission |  |  |  |  |  |  |  |
| *2012* | 1.000 |  |  |  | 1.000 |  |  |
| 2013 | 1.020 | (0.972-1.070) | 0.421 |  | 0.996 | (0.981-1.011) | 0.588 |
| 2014 | 1.000 | (0.949-1.055) | 0.996 |  | 1.045 | (1.028-1.063) | 0.000 |
| 2015 | 0.963 | (0.914-1.015) | 0.161 |  | 1.074 | (1.056-1.092) | 0.000 |
| Month of admission |  |  |  |  |  |  |  |
| *January* | 1.000 |  |  |  | 1.000 |  |  |
| February | 1.040 | (0.960-1.128) | 0.335 |  | 0.989 | (0.964-1.015) | 0.409 |
| March | 1.015 | (0.937-1.100) | 0.715 |  | 1.004 | (0.978-1.030) | 0.770 |
| April | 0.950 | (0.874-1.032) | 0.222 |  | 0.995 | (0.969-1.021) | 0.689 |
| May | 1.015 | (0.934-1.104) | 0.719 |  | 1.007 | (0.981-1.033) | 0.625 |
| June | 1.041 | (0.958-1.130) | 0.345 |  | 1.014 | (0.988-1.040) | 0.309 |
| July | 0.980 | (0.902-1.065) | 0.638 |  | 1.009 | (0.984-1.036) | 0.477 |
| August | 1.094 | (1.008-1.188) | 0.032 |  | 0.987 | (0.961-1.013) | 0.314 |
| September | 1.046 | (0.963-1.136) | 0.282 |  | 1.008 | (0.982-1.034) | 0.568 |
| October | 0.982 | (0.904-1.066) | 0.664 |  | 0.995 | (0.970-1.021) | 0.723 |
| November | 0.966 | (0.888-1.050) | 0.410 |  | 0.996 | (0.970-1.022) | 0.753 |
| December | 0.983 | (0.905-1.067) | 0.680 |  | 0.988 | (0.963-1.015) | 0.377 |
| Admission Type |  |  |  |  |  |  |  |
| *medical* | 1.000 |  |  |  | 1.000 |  |  |
| unscheduled surgery | 0.688 | (0.653-0.724) | 0.000 |  | 0.873 | (0.857-0.890) | 0.000 |
| scheduled surgery | 0.508 | (0.468-0.551) | 0.000 |  | 1.204 | (1.183-1.226) | 0.000 |
| surgery unspecified | 1.282 | (1.151-1.428) | 0.000 |  | 0.932 | (0.903-0.963) | 0.000 |
| unknown | 1.585 | (1.411-1.782) | 0.000 |  | 1.257 | (1.220-1.295) | 0.000 |
| Weekday of admission |  |  |  |  |  |  |  |
| Monday | 1.061 | (0.995-1.131) | 0.070 |  | 0.948 | (0.929-0.968) | 0.000 |
| Tuesday | 0.989 | (0.929-1.054) | 0.742 |  | 0.979 | (0.960-0.998) | 0.030 |
| *Wednesday* | 1.000 |  |  |  | 1.000 |  |  |
| Thursday | 0.933 | (0.875-0.994) | 0.033 |  | 1.003 | (0.984-1.022) | 0.779 |
| Friday | 1.009 | (0.946-1.076) | 0.787 |  | 1.068 | (1.047-1.090) | 0.000 |
| Saturday | 1.102 | (1.030-1.179) | 0.005 |  | 1.004 | (0.980-1.029) | 0.748 |
| Sunday | 1.087 | (1.015-1.164) | 0.017 |  | 0.931 | (0.908-0.954) | 0.000 |
| Weekday of event |  |  |  |  |  |  |  |
| Monday | 0.986 | (0.924-1.052) | 0.673 |  | 0.993 | (0.972-1.014) | 0.503 |
| Tuesday | 1.021 | (0.959-1.088) | 0.515 |  | 1.011 | (0.991-1.031) | 0.276 |
| *Wednesday* | 1.000 |  |  |  | 1.000 |  |  |
| Thursday | 1.040 | (0.976-1.108) | 0.226 |  | 0.975 | (0.956-0.994) | 0.010 |
| Friday | 1.040 | (0.975-1.110) | 0.228 |  | 1.004 | (0.984-1.024) | 0.694 |
| Saturday | 0.992 | (0.929-1.060) | 0.814 |  | 0.631 | (0.617-0.646) | 0.000 |
| Sunday | 0.890 | (0.831-0.952) | 0.001 |  | 0.552 | (0.539-0.566) | 0.000 |

# S11 Main Analysis without censoring after 30 days

|  | Death in ICU | | |  | ICU Discharge | | |
| --- | --- | --- | --- | --- | --- | --- | --- |
|  | HR | 95% CI | p |  | HR | 95% CI | p |
| SAPS 3 [per 10 points] | 1.901 | (1.883-1.920) | 0.000 |  | 0.686 | (0.683-0.689) | 0.000 |
| Year of admission |  |  |  |  |  |  |  |
| *2012* | 1.000 |  |  |  | 1.000 |  |  |
| 2013 | 1.008 | (0.962-1.057) | 0.734 |  | 0.993 | (0.978-1.007) | 0.333 |
| 2014 | 0.980 | (0.930-1.032) | 0.436 |  | 1.047 | (1.030-1.065) | 0.000 |
| 2015 | 0.920 | (0.874-0.969) | 0.002 |  | 1.082 | (1.064-1.100) | 0.000 |
| Month of admission |  |  |  |  |  |  |  |
| *January* | 1.000 |  |  |  | 1.000 |  |  |
| February | 1.075 | (0.994-1.163) | 0.071 |  | 0.978 | (0.953-1.003) | 0.085 |
| March | 1.014 | (0.938-1.097) | 0.725 |  | 0.994 | (0.969-1.020) | 0.652 |
| April | 0.965 | (0.890-1.047) | 0.391 |  | 0.998 | (0.973-1.024) | 0.876 |
| May | 1.015 | (0.936-1.101) | 0.717 |  | 1.008 | (0.982-1.034) | 0.564 |
| June | 1.016 | (0.937-1.101) | 0.706 |  | 1.008 | (0.982-1.034) | 0.555 |
| July | 0.971 | (0.895-1.053) | 0.476 |  | 1.014 | (0.989-1.040) | 0.284 |
| August | 1.087 | (1.003-1.177) | 0.043 |  | 0.987 | (0.962-1.013) | 0.336 |
| September | 1.020 | (0.941-1.106) | 0.633 |  | 1.002 | (0.977-1.028) | 0.878 |
| October | 0.957 | (0.882-1.037) | 0.283 |  | 1.003 | (0.978-1.028) | 0.831 |
| November | 1.000 | (0.921-1.085) | 0.994 |  | 0.997 | (0.972-1.023) | 0.843 |
| December | 0.986 | (0.909-1.069) | 0.729 |  | 0.987 | (0.962-1.013) | 0.342 |
| Admission Type |  |  |  |  |  |  |  |
| *medical* | 1.000 |  |  |  | 1.000 |  |  |
| unscheduled surgery | 0.769 | (0.732-0.808) | 0.000 |  | 0.963 | (0.945-0.982) | 0.000 |
| scheduled surgery | 0.452 | (0.418-0.488) | 0.000 |  | 1.203 | (1.182-1.225) | 0.000 |
| surgery unspecified | 1.316 | (1.184-1.463) | 0.000 |  | 0.929 | (0.899-0.958) | 0.000 |
| unknown | 1.348 | (1.202-1.512) | 0.000 |  | 1.146 | (1.113-1.180) | 0.000 |
| Weekday of admission |  |  |  |  |  |  |  |
| Monday | 1.059 | (0.995-1.127) | 0.071 |  | 0.941 | (0.922-0.960) | 0.000 |
| Tuesday | 0.982 | (0.924-1.045) | 0.570 |  | 0.979 | (0.961-0.998) | 0.028 |
| *Wednesday* | 1.000 |  |  |  | 1.000 |  |  |
| Thursday | 0.955 | (0.897-1.016) | 0.144 |  | 1.016 | (0.997-1.035) | 0.109 |
| Friday | 1.064 | (1.000-1.132) | 0.050 |  | 1.068 | (1.047-1.090) | 0.000 |
| Saturday | 1.160 | (1.086-1.239) | 0.000 |  | 0.986 | (0.962-1.009) | 0.229 |
| Sunday | 1.118 | (1.046-1.195) | 0.001 |  | 0.915 | (0.893-0.938) | 0.000 |
| Weekday of event |  |  |  |  |  |  |  |
| Monday | 0.977 | (0.917-1.041) | 0.473 |  | 1.014 | (0.993-1.035) | 0.196 |
| Tuesday | 1.018 | (0.958-1.083) | 0.564 |  | 1.019 | (0.999-1.039) | 0.057 |
| *Wednesday* | 1.000 |  |  |  | 1.000 |  |  |
| Thursday | 1.010 | (0.950-1.074) | 0.746 |  | 0.971 | (0.953-0.990) | 0.003 |
| Friday | 0.984 | (0.925-1.048) | 0.621 |  | 0.992 | (0.973-1.012) | 0.441 |
| Saturday | 0.911 | (0.855-0.972) | 0.005 |  | 0.620 | (0.606-0.634) | 0.000 |
| Sunday | 0.835 | (0.782-0.893) | 0.000 |  | 0.549 | (0.536-0.563) | 0.000 |
